# Halofuginone exerts broad-spectrum cytotoxic effects by regulating p-eIF2α-S100A8/A9-calcium signaling, inhibiting global protein synthesis, and reversing the resistance of idarubicin in acute myeloid leukemia

**DOI:** 10.1186/s13020-025-01278-9

**Published:** 2026-01-06

**Authors:** Liuzhi Shi, Min Zhao, Chen Meng, Min Li, Xuanyu Yu, Shixin Zhang, Shirui Yu, Xinyao Chen, Bin Zhou, Chongyun Xing, Jinjun Jia, Jingying Zhou, Shenmeng Gao

**Affiliations:** 1 Department of Clinical Laboratory, The First Affiliated Hospital of Wenzhou Medical University, 1 Xuefubei Street, Ouhai District, Wenzhou, 325000 Zhejiang Province China; 2 Medical Research Center, The First Affiliated Hospital of Wenzhou Medical University, 1 Xuefubei Street, Ouhai District, Wenzhou, 325000 Zhejiang Province China; 3 Department of Hematology, The First Affiliated Hospital of Wenzhou Medical University, 1 Xuefubei Street, Ouhai District, Wenzhou, Zhejiang Province China; 4 Alberta Institute Wenzhou Medical University, Ouhai District, Wenzhou, Zhejiang Province China; 5 Department of Pediatrics, First Affiliated Hospital of Ningbo University, 59 Liuting Road, Ningbo, 315000 Zhejiang Province China; 6 The Key Laboratory of Pediatric Hematology and Oncology Diseases of Wenzhou, The Second Affiliated Hospital and Yuying Children’s Hospital of Wenzhou Medical University, Wenzhou, Zhejiang China

**Keywords:** Halofuginone, Acute myeloid leukemia, S100A8, S100A9

## Abstract

**Background:**

Acute myeloid leukemia (AML) is a heterogeneous hematologic malignancy with poor overall survival (OS). Resistance to chemotherapeutic drugs such as idarubicin (IDA) remains a major cause of treatment failure. This study investigated the anti-leukemic activity of halofuginone (HF) a synthetic derivative of the natural compound from hydrangea Dichroa febrifuge and its potential to overcome IDA resistance in AML cells.

**Methods:**

Apoptosis, proliferation, cell cycle, and colony formation were assessed in AML cells treated with HF. RNA sequencing (RNA-seq) was performed to identify the potential molecular targets of HF. The anti-leukemic efficacy of HF was further assessed in NOD/SCID-IL2Rγ (NSG) mice xenografted with human relapsed/refractory (R/R) AML samples.

**Results:**

HF treatment significantly inhibited cell proliferation, reduced colony formation, and induced apoptosis in AML cells. By RNA-seq analysis, S100A8 and S100A9 (S100A8/A9) were identified as potential targets of HF, and HF treatment markedly suppressed their expression. Overexpression of S100A8/A9 abrogated the anti-leukemic effects of HF, indicating that S100A8/A9 are critical mediators of HF activity. Mechanistically, HF activated the amino acid starvation response (AAR), leading to phosphorylation of eukaryotic translation initiation factor 2 subunit alpha (p-eIF2α), subsequent downregulation of S100A8/A9, and elevation of cytoplasmic Ca^2^⁺ levels. Knockdown of eIF2α prevented HF-induced downregulation of S100A8/A9, confirming that HF regulates S100A8/A9 expression via the eIF2α pathway. Furthermore, HF treatment inhibited global protein synthesis, enhanced the cytotoxicity of chemotherapeutic drugs, and reversed IDA resistance by suppressing S100A8/A9 expression. Finally, HF inhibits leukemic infiltration and extended OS in MLL-AF9-transduced AML mice and enhanced IDA-induced anti-leukemic effects in R/R AML-xenografted NSG mice model.

**Conclusions:**

These findings reveal that HF exerts anti-leukemic effects by modulating the p-eIF2α–S100A8/A9–Ca^2^⁺ signaling axis in AML cells. HF represents a promising therapeutic candidate for AML, particularly for patients with IDA-resistant disease.

**Supplementary Information:**

The online version contains supplementary material available at 10.1186/s13020-025-01278-9.

## Introduction

Acute myeloid leukemia (AML) is a highly heterogeneous hematologic malignancy with diverse clinical and molecular subtypes [1]. This heterogeneity results in different clinical outcomes. For instance, the combination of arsenic trioxide and all-trans retinoic acid is highly effective in acute promyelocytic leukemia [2], but not in other AML subtypes. In AML, normal hematopoietic stem and progenitor cells (HSPCs) acquire leukemia stem cell properties through uncontrolled proliferation and impaired differentiation, driven by cumulative genetic and epigenetic alterations [3, 4]. Although standard chemotherapy, immunotherapy, and bone marrow transplantation (BMT) have improved overall survival (OS), relapse and chemoresistance remain major obstacles for most AML patients. Therefore, developing novel therapeutic strategies to overcome both primary and acquired resistance is an urgent priority [5].

Halofuginone (HF), a synthetic derivative of the natural compound *Dichroa febrifuga*, was developed to reduce the toxicity of its parent compound febrifugine [6]. HF has been approved by the U.S. Food and Drug Administration as a feed additive for the prevention of coccidiosis in poultry [7], suggesting a relatively low toxicity profile. Traditionally used in Chinese medicine to treat malaria [8], HF has recently been shown to directly bind and inhibit glutamyl-prolyl-tRNA synthetase (EPRS) [9], thereby reducing proline (Pro) availability and activating the amino acid starvation response (AAR) [10]. Experimentally, HF has demonstrated anti-tumor properties, including inhibition of angiogenesis, metastasis, and proliferation. In acute promyelocytic leukemia (APL), HF suppresses leukemia growth and angiogenesis via inhibition of SMAD2 signaling [11]. However, the precise role and molecular mechanism of HF-induced anti-leukemic effects remain unclear.

Eukaryotic translation initiation factor 2 subunit alpha (eIF2α) plays a rate-limiting role in translation initiation. Dysregulated eIF2α activity contributes to malignant transformation, underscoring its importance in regulating cell proliferation and survival [12]. HF-induced AAR by inhibiting EPRS facilitates the phosphorylation of eIF2α at serine 51. Such phosphorylation converts eIF2 to a competitive inhibitor of eIF2B, thereby reducing translation initiation and protein synthesis [12, 13]. Moreover, AAR-induced p-eIF2α modulates the transcription of genes involved in RNA binding and RNA splicing [14]. Therefore, we assumed that HF regulates p-elF2α by inducing AAR. However, whether HF-induced p-eIF2α mediates its anti-leukemic effects has not been fully elucidated.

S100A8 and S100A9 (S100A8/A9), members of the evolutionarily conserved S100 leukocyte protein family, are Ca^2+^-binding proteins [15]. These proteins form heterodimers, which bind to Ca^2+^ to form more stable heterotetramers, known as calprotectin. S100A8/A9 heterotetramers transfer Ca^2+^ signals in the cytoplasm to regulate cellular activities, such as inflammation, apoptosis, and differentiation [16]. In addition, secreted S100A8/A9 interact with receptors such as Toll-like receptor 4 (TLR4) and receptor for advanced glycation end-product (RAGE) to influence differentiation and proliferation [17, 18]. Elevated S100A8/A9 levels have been observed in AML patient samples [19] and correlate with poorer prognosis [20, 21]. Elevated levels of S100A8/A9 are also associated with resistance to glucocorticoid [22] and Gilteritinib [23]. Given their role in enhancing the proliferation, survival, and resistance to chemotherapy [24], S100A8/A9 represent promising therapeutic targets in AML. However, whether HF decreases S100A8/A9 by inducing AAR-mediated p-eIF2α accumulation is not determined in AML cells.

Here, we report that HF exerts potent anti-leukemic effects by modulating the p-eIF2α-S100A8/A9-Ca^2^⁺ signaling axis and suppressing global protein synthesis. HF enhances the cytotoxicity of chemotherapeutic agents and reverses idarubicin (IDA) resistance through downregulation of S100A8/A9 expression. Collectively, these findings indicate that HF represents a promising therapeutic candidate for AML, particularly for patients with relapsed/refractory (R/R) disease.

## Methods and materials

### Leukemic cell lines and primary AML blasts

Human leukemic cell lines obtained from American Type Culture Collection (ATCC, Manassas, VA, USA) and Deutsche Sammlung von Mikroorganismen und Zellkulturen (DSMZ, Braunschweig, Germany) were cultured in complete RPMI 1640 medium (Invitrogen, Carlsbad, CA, USA) supplemented with 10% fetal bovine serum (Invitrogen), 100 U/mL penicillin, and 100 mg/mL streptomycin in a humidified incubator with 5% CO_2_ at 37 °C. Human bone marrow (BM) mononuclear cells were isolated from de novo or R/R AML patients using Ficoll-Paque gradient (GE Healthcare Bio-Sciences, Pittsburgh, PA, USA) and cultured in StemSpan SFEM medium supplemented with human recombinant stem cell factor (SCF), interleukin-3 (IL-3), and interleukin-6 (IL-6), at 10 ng/mL each. All procedures involving human participants were conducted in accordance with the ethical standards of the Ethics Committee of the First Affiliated Hospital of Wenzhou Medical University and the Declaration of Helsinki. Informed consent was obtained from AML patients.

### RNA extraction and quantitative real-time PCR (qRT-PCR)

Total RNA was extracted using TRIzol (Invitrogen) according to the manufacturer's instructions. Leukemic cells were lysed by TRIzol and incubated at room temperature for 15 min. Chloroform (0.2 mL per sample) was added, and the mixture was thoroughly mixed. After centrifugation at 12,000 × rpm for 15 min at 4 °C, the aqueous phase was transferred, and an equal volume of isopropanol was added. RNA was precipitated after centrifugation at 7,500 × rpm for 5 min, and washed with 75% ethanol, and dissolved in TE buffer. After RNA concentration was measured by a DS-11 spectrophotometer (DeNovix, Wilmington, DE, USA), total RNA was used as a template for cDNA synthesis with the Primescript™ RT Master Mix (Takara) on the ABI 7500 real-time PCR system (Applied Biosystems, Carlsbad, CA, USA). cDNA was used as a template to amplify gene expression using TB Green Premix Ex Taq™ (Takara). GAPDH and β-actin served as endogenous controls for human and murine samples, respectively. Each sample was run in triplicate. Relative expression was calculated using the 2^−ΔΔCT^ method. The primer sequences are indicated in Table S1.

### Plasmid production

The wild-type coding sequences (CDS) of *S100A8* and *S100A9* were amplified from cDNA of a healthy donor and inserted into the lentiviral vector pLVX-puromycin (Sigma-Aldrich). Gene-specific short hairpin RNAs (shRNAs) targeting *S100A8* and *S100A9* were designed and cloned into vector pLKO.1-puromycin and pLKO.1-green fluorescent protein (GFP) (Sigma-Aldrich) vectors, respectively. splashRNAs for eIF2α were designed and cloned into miRNA-based vectors (http://splashrna.mskcc.org/) [25]. Control shRNA is a nonfunctional construct. All primer sequences are provided in Table S1, and the constructs were confirmed by DNA sequencing.

### Virus production and cell infection

These procedures are performed as previously documented [26]. Briefly, 293 T cells (4 × 10^6^) were plated in 10 cm dishes 24 h before transfection. For lentivirus production, expression vectors and packaging plasmids (PSPA2 and MD2G) were transfected into 293 T cells using polyethylenimine (PEI, Sigma-Aldrich). Lentivirus supernatants were harvested and filtered through a low protein-binding-polysulfone filter (0.45 μm, Millipore, Billerica, MA, USA) 48 and 72 h after the transfection. Leukemic cells were suspended in viral supernatant in six-well plates with 8 μg/mL polybrene (Sigma-Aldrich) and centrifuged at 2,000 × g for 2 h. GFP^+^ cells were sorted by flow cytometry for the following analysis. Alternatively, puromycin (1 μg/mL) was added to the medium to select positive clones for 2–4 days.

### Mice housing

All laboratory C57/B6 mice and NSG mice (Shanghai Model Organisms Center, Shanghai, China) were housed at 25 ℃ with 12 h light and 12 h dark cycles and maintained under standard housing conditions in the animal core facility of the First Affiliated Hospital of Wenzhou Medical University. All procedures involving mice were approved by the Animal Care and Use Committee of the First Affiliated Hospital of Wenzhou Medical University (WYYY-AEC-2023–017).

### Engraftment of NOD/SCID‑IL2Rγ mice (NSG)

Male NSG mice (8 weeks old, Shanghai Model Organisms Center) were intraperitoneally injected with busulfan (30 mg/kg, Sigma) one day before transplantation. Each NSG mouse was xenografted with MOLM-13 cells (1 × 10^6^). Mice were randomly divided into the following groups: Ctrl (n = 6), HF (0.25 mg/kg, n = 9), or HF (0.5 mg/kg, n = 9) [27]. In addition, primary leukemia cells (1 × 10^6^) from R/R AML patients were injected via the tail vein. Mice were randomly assigned to one of the following treatment groups: Ctrl (n = 6 for AML 6 and 11), HF (0.5 mg/kg, n = 7 for AML 6 and n = 8 for AML 11), IDA (1.5 mg/kg, n = 6 for AML 6 and 11), or IDA + HF (n = 8 for AML 6 and n = 9 for AML 11). HF and IDA were dissolved in a solution containing 10% DMSO, 45% PEG300, and 45% Saline. Control mice received the placebo (10% DMSO, 45% PEG300, and 45% Saline). Treatment was administered once daily for 10 days, starting approximately three weeks after BMT, when the frequency of human CD45 (hCD45) in blood exceeded 1%. OS time was measured from the first day of transplantation until death.

### MLL-AF9-induced murine leukemic model for in vivo treatment

MSCV-GFP-internal ribosome entry site (IRES)-MLL-AF9 and the packaging plasmid pCL-ECO were transfected into 293 T cells, and retrovirus supernatants were collected 48 and 72 h post-transfection. Mouse Lineage (Lin^−^) cells were isolated from normal C57/B6 mice (Shanghai Model Organisms Center) after 5-fluorouracil (5-flu) treatment. These cells were transduced with MSCV-GFP-IRES-MLL-AF9-retrovirus by spinoculation at 2,000 × rpm for 2 h [28]. After centrifugation and removal of the supernatant, Lin^−^ cells were cultured in StemSpan SFEM (Stemcell Technologies) supplemented with murine SCF (100 ng/mL, PeproTech), TPO (100 ng/mL, PeproTech), and FLT3 ligand (100 ng/mL, PeproTech) in a humidified 37 °C incubator with 5% CO_2_ for 48 h. GFP^+^ cells were sorted and transplanted into lethally irradiated C57BL/6 J mice (Beijing Vital River Laboratory). Recipient mice were humanely sacrificed by CO_2_ inhalation when they showed signs of typical leukemic symptoms, such as paralysis or labored breathing. BM mononuclear cells were isolated from the femur and tibia, and GFP^+^ cells were sorted by flow cytometry and xenografted into lethally irradiated C57BL/6 J mice. These mice were randomly divided into two groups: Control (Ctrl, n = 7) and HF (0.5 mg/kg, n = 7). Ctrl mice received the placebo (10% DMSO + 45% PEG300 + 45% Saline). HF was dissolved in a solution containing 10% DMSO, 45% PEG300, and 45% Saline. Treatment was administered once daily for 10 days, starting approximately two weeks after BMT, when the frequency of GFP^+^ cells in the blood exceeded 1%. OS time was determined from the first day of transplantation until death.

### Surface sensing of translation (SUnSET) assay

AML cells were incubated with puromycin (10 μg/mL) for 10 min at 37 °C incubator with 5% CO_2_. AML cells were collected and centrifuged at 1000×RPM for 5 min to remove the supernatant and washed with 10% RPMI 1640 medium containing 10% FBS twice. AML cells were re-cultured in RPMI 1640 medium containing 10% FBS in a humidified 37 °C incubator with 5% CO_2 _for 50 min, and were subjected to Western blot using an anti-puromycin antibody (1:5000, MABE343, Millipore).

### Data availability

All RNA-seq data are publicly available on the Gene Expression Omnibus under accession numbers GSE 303946, GSE 303795. Additional methods are described in Supplemental Methods.

Apoptosis assay, mitochondrial membrane potential (MMP) assay, colony-forming unit assay, cell cycle analysis, CCK-8, viability assay, Western blot, cytoplasmic Ca^2+^ level assay, construction of IDA-resistance cells, reagents, enrolled AML patients, and RNA sequencing (RNA-seq) analysis please see supplemental materials.

### Statistical analysis

Data are presented as mean ± standard deviation (SD). Generally, a two-tailed Student's t-test was used to evaluate differences between two groups. For comparisons of multiple groups, a two-way analysis of variance (ANOVA) with Tukey's test was applied. Kaplan–Meier survival curves were produced using GraphPad Prism 9.0 (GraphPad Software Inc., USA) to analyze OS time, and *P* values were calculated by the log-rank test. The chi-square test or Fisher's exact test was performed for categorical covariates. All statistical analyses were performed using GraphPad Prism 9.0 (GraphPad Software Inc., USA). Differences are considered significant when the *P* value is < 0.05.

## Results

### Anti-leukemic effects of HF in AML cells

**Fig. 1:**
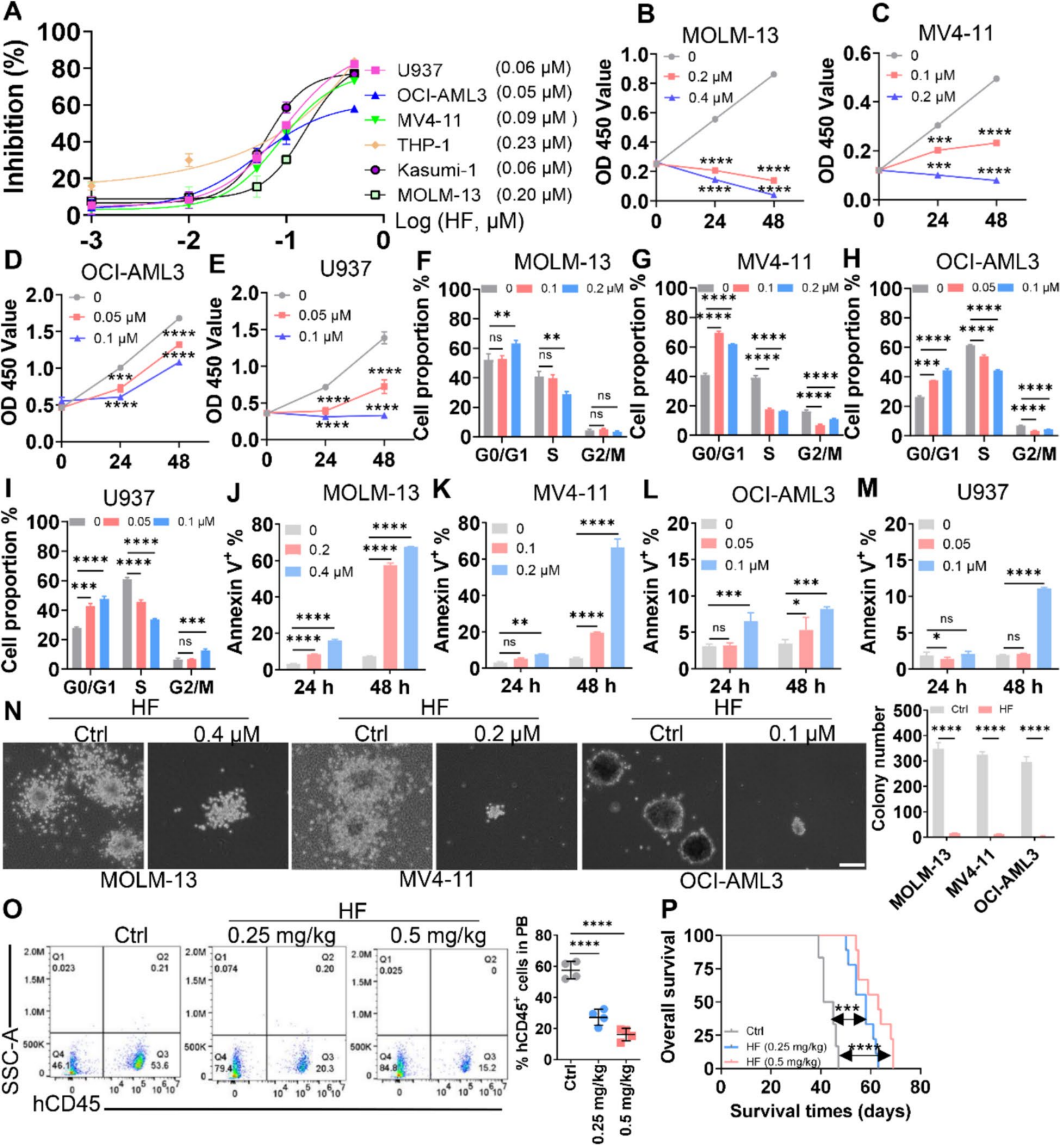
Cytotoxic effects of HF on leukemic cells. **A** Growth inhibition was assessed by CCK-8 assay in six AML cell lines treated with various concentrations of HF for 24 h, and the IC50 values were calculated. **B**–**E** Cell proliferation was evaluated by the CCK-8 assay in AML cell lines incubated with different concentrations of HF for 24 and 48 h. **F**–**I** Cell cycle distribution was azalyzed by PI staining in four AML cell lines treated with different concentrations of HF for 24 h. Statistical analyses of the G2/M, S, and G0/G1 phases are shown. **J**–**M** Apoptosis was measured by Annexin V/7-AAD staining in four AML cell lines treated with different concentrations of HF for 24 and 48 h. **N** AML cells (2 × 10^3^/dish) were treated with the indicated concentrations of HF and plated in methylcellulose medium for 10 days to assess colony number. Representative pictures (left) and statistical analysis of colony number (right) are shown. Bar scales represent 100 μm. **O** and **P** MOLM-13 cells were xenografted into NSG mice and treated with a placebo as control (Ctrl), HF (0.25 mg/kg), or HF (0.5 mg/kg). The percentage of human CD45 (hCD45) in PB (**O**) and OS (**P**) were assessed in NSG mice treated with Ctrl (n = 6), HF (0.25 mg/kg, n = 9), or HF (0.5 mg/kg, n = 9). Representative plots (left) and statistical analysis of hCD45 (right) are shown. n = 3 or more independent biological replicates. *P < 0.05; **P < 0.01; ***P < 0.001; ****P < 0.0001 versus untreated cells. ns: not significant

To systematically explore the anti-leukemic effects of HF, we first determined the IC50 of HF in several AML cell lines, including MOLM-13, Kasumi-1, and MV4-11. Most AML cell lines demonstrated high sensitivity to HF treatment with low IC50 values (0.05–0.2 μM) (Fig. 1A). Therefore, cell lines with different genetic backgrounds, including MOLM-13, MV4-11, OCI-AML3, and U937 cells, were treated with low (IC50) and high (2 × IC50) concentrations of HF for subsequent analysis. HF significantly suppressed cell proliferation in a time-dependent manner (Fig. 1B–E) and induced cell cycle arrest at the G0/G1 phase (Figs. 1F–I and S1A–D). Remarkably, HF also induced apoptosis in a time and concentration-dependent manner, as evidenced by the increased Annexin V^+^ cells (Fig. 1J–M and S2A–D) and upregulated protein levels of cleaved PARP and caspase-3 (Fig. S2E–H). At higher concentration, HF almost completely inhibited colony formation (Fig. 1N). Given the observed apoptotic effects, we next assessed mitochondrial integrity. JC-1 staining revealed a marked loss of mitochondrial membrane potential (MMP) following HF exposure (Fig. S3A, B), indicating mitochondrial dysfunction. To further investigate the anti-leukemic effects of HF in vivo, we xenografted MOLM-13 cells into NSG mice and treated them with or without HF. HF treatment significantly reduced the percentage of MOLM-13 (identified by hCD45) in peripheral blood (PB) (Fig. 1O) and extended the OS of NSG mice compared with Ctrl treatment (Fig. 1P). These findings indicate that HF exhibits broad-spectrum cytotoxic effects in AML cells, including inhibition of proliferation and colony formation, as well as induction of apoptosis and cell cycle arrest.

### HF decreases S100A8/A9 expression in AML cells

**Fig. 2:**
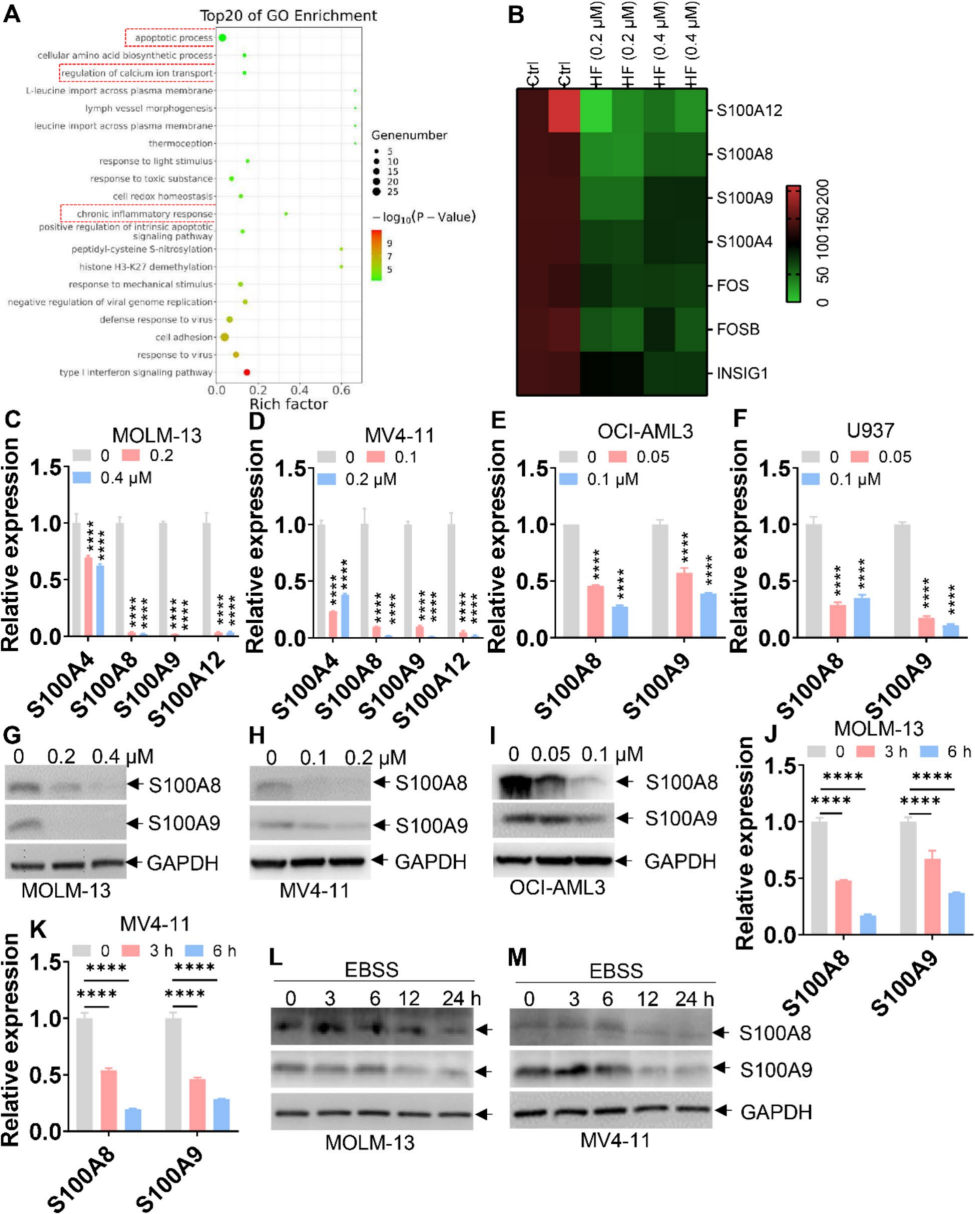
HF and amino acid and serum starvation (EBSS) decrease S100A8 and S100A9 expressions. **A** and **B** MOLM-13 cells were treated with HF (0.2 or 0.4 μM) for 24 h, and total RNA was extracted for RNA-Seq. Gene Ontology analysis (GO, A) and heatmap analysis (**B**) are shown. **C** and **D **The mRNA expressions of *S100A4*, *A8*, *A9*, and *A12* were measured in MOLM-13 and MV4-11 cells treated with various concentrations of HF for 24 h. **E** and **F** The mRNA expressions of *S100A8*/*A9* were measured in OCI-AML3 and U937 cells treated with different concentrations of HF for 24 h. **G**–**I** The protein expressions of *S100A8*/*A9* were measured in MOLM-13, MV4-11, and OCI-AML3 cells treated with different concentrations of HF for 24 h. **J** and **K** The mRNA expressions of *S100A8*/*A9* were measured in MOLM-13 and MV4-11 cells under EBSS condition for 3 and 6 h. (**L** and **M**) The protein expressions of S100A8/A9 were measured in MOLM-13 and MV4-11 cells under EBSS conditions for the indicated times. n = 3 or more independent biological replicates. ****P < 0.0001 versus untreated cells

To identify the potential downstream targets of HF, MOLM-13 cells were treated with 0.2 μM HF for 24 h and analyzed for RNA-seq. Gene ontology (GO) analysis revealed significant enrichment in pathways associated with apoptosis and calcium ion transport (Fig. 2A). Notably, several *S100* family genes—including *S100A4*, *S100A8*, *S100A9*, and *S100A12*—were among the most downregulated following HF exposure (Fig. 2B). qRT-PCR confirmed that the mRNA levels of these *S100* genes were significantly reduced in HF-treated AML cells (Fig. 2C, D). As reported that S100A8 and S100A9 (S100A8/A9) are highly expressed in AML cells and facilitate leukemogenesis [29, 30], we focused subsequent analyses on this gene pair. Consistently, HF decreased the transcript levels of *S100A8*/*A9* in OCI-AML3 and U937 cells (Fig. 2E, F) and substantially reduced protein levels of S100A8/A9 in three AML cell lines (Fig. 2G–I).

Since HF inhibits prolyl-tRNA synthetase activity and mimics amino acid starvation [9, 31], we next examined *S100A8/A9* expression under amino acid and serum starvation (EBSS). As expected, the transcript (Fig. 2J, K) and protein levels (Fig. 2L, M) of S100A8/A9 were significantly downregulated in AML cells cultured in EBSS condition, recapitulating the HF effect.

### S100A8/A9 expression levels are elevated in AML and predict poor prognosis

To explore the clinical relevance of S100A8/A9 in AML, we first analyzed S100A8/A9 expression using the Gene Expression Profiling Interactive Analysis (GEPIA) database [32]. AML patient samples had higher levels of S100A8/A9 than NC samples (Fig. S4A, B). High levels of *S100A8/A9* were associated with inferior OS in AML patients from the BEAT AML database (Fig. S4C, D). *S100A8* expression showed a strong positive correlation with *S100A9* (Fig. S4E).

To further validate these findings, we analyzed *S100A8/A9* expression in 94 AML patient samples received standard induction regimen (Table S2). Consistently, patients with higher *S100A8/A9* expression (above median) exhibited significantly shorter OS (Fig. S5A, B) and relapse-free survival (RFS) (Fig. S5C, D), as well as lower complete remission (CR) rates (Fig. S5E, F), compared with those with lower expression levels (below median). These results indicate that *S100A8/A9* upregulation correlates with poor clinical outcomes in AML patients.

### S100A8/A9-Ca^2+^ signaling is a functional target of HF

**Fig. 3:**
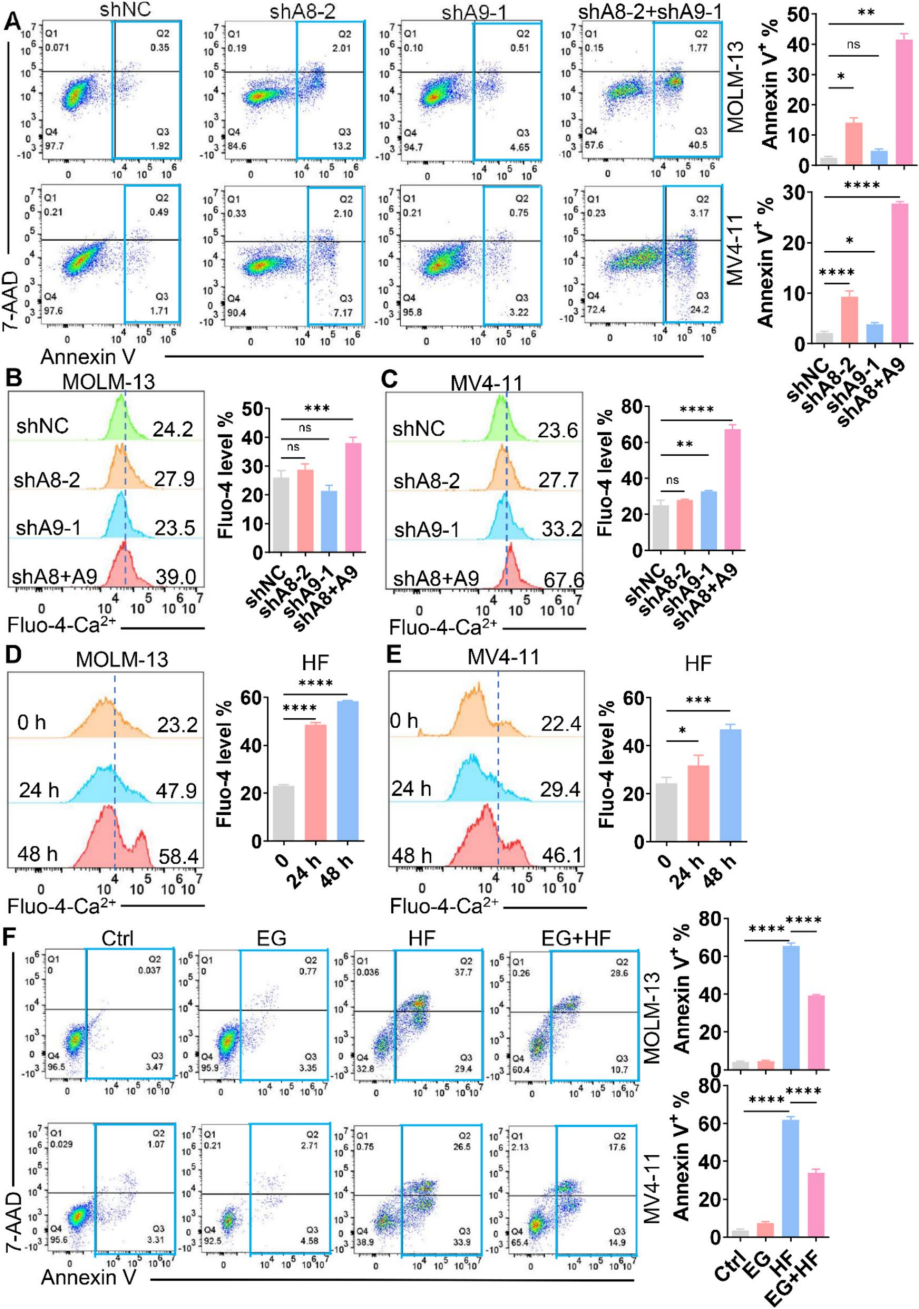
HF exerts cytotoxic effects on AML cells by regulating S100A8/A9-Ca^2+^ signaling pathway. **A** Apoptosis was measured in MOLM-13 and MV4-11 cells, which were transduced with shNC, shS100A8-2 (shA8-2), shS100A9-1 (shA9-1), or shA8 + shA9 for 48 h and treated with puromycin (1 μg/mL) for an additional 48 h. The representative plots of Annexin V/7-AAD staining (left) and quantification of Annexin V^+^ cells (right) are shown. **B** and **C** Cytoplasmic Ca^2+^ levels were measured in MOLM-13 and MV4-11 cells transduced with shNC, shA8-2, shA9-1, or shA8 + shA9 for 48 h, followed by puromycin (1 μg/mL) treatment for an additional 48 h. Representative plots (left) and quantification of cytoplasmic Ca^2+^ levels (right) are shown. **D** and **E** Cytoplasmic Ca^2+^ levels were measured in MOLM-13 and MV4-11 cells treated with HF (0.4 μM for MOLM-13; 0.2 μM for MV4-11) for 24 and 48 h. Representative plots (left) and quantification of Ca^2+^ levels (right) are shown. **F** Apoptosis was measured in MOLM-13 and MV4-11 cells treated with Ctrl, Ca^2+^ inhibitor EGTA (EG, 0.8 mM), HF (0.4 μM for MOLM-13; 0.2 μM for MV4-11), or EG + HF for 48 h. The representative plots (left) and quantification of Annexin V^+^ cells (right) are shown. n = 3 or more independent biological replicates. *P < 0.05; **P < 0.01; ***P < 0.001; ****P < 0.0001. ns: not significant

To investigate the functional role of *S100A8/A9* in AML, we transduced AML cells with shRNAs targeting *S100A8* or *S100A9*. Knockdown efficiency was confirmed by qRT-PCR and Western blot (Fig. S6A–D). Silencing either gene suppressed AML cell proliferation (Fig. S6E–H). Given the high sequence similarity between *S100A8* and *S100A9*, we further confirmed the specificity of each shRNA: *S100A8*-specific shRNA selectively reduced *S100A8* expression with minimal effect on *S100A9* (Fig. S7A, B), while *S100A9*-specific shRNA selectively downregulated *S100A9* without altering *S100A8* levels (Fig. S7C, D). Simultaneous knockdown of *S100A8* and *S100A9* significantly enhanced apoptosis compared with individual knockdowns (Fig. 3A), indicating a synergistic pro-survival role of *S100A8/A9* in AML. Because HF downregulates *S100A8/A9* expression, we performed rescue experiments in HF-treated AML cells overexpressing *S100A8/A9*. Overexpression was confirmed at both mRNA and protein levels (Fig. S8A–C). Importantly, enforced *S100A8/A9* expression markedly attenuated HF-induced apoptosis (Fig. S8D), suggesting that *S100A8/A9* are critical downstream mediators of HF activity.

Since intracellular heterodimeric S100A8/A9 binds to Ca^2+^ to form heterotetramer complex [16], we next examined whether S100A8/A9 knockdown affects cytoplasmic Ca^2+^ levels. Knockdown of both S100A8 and S100A9 significantly increased cytoplasmic Ca^2+^ levels more than a single knockdown of S100A8 or S100A9 (Fig. 3B, C). Since HF treatment decreased S100A8/A9 expression and S100A8/A9 knockdown triggered cytoplasmic Ca^2+^ level, we examined whether HF also increased cytoplasmic Ca^2+^ levels. As anticipated, HF treatment significantly increased cytoplasmic Ca^2+^ level in a time-dependent manner (Fig. 3D, E). Importantly, Ca^2+^ inhibitors EGTA and BAPTA-AM substantially rescued HF-induced apoptosis in AML cells (Fig. 3F, S9A). These results demonstrate that HF exerts anti-leukemic effects through the S100A8/A9-Ca^2+^ signaling pathway.

### HF decreases S100A8/A9 expression by triggering the accumulation of p-eIF2α

**Fig. 4:**
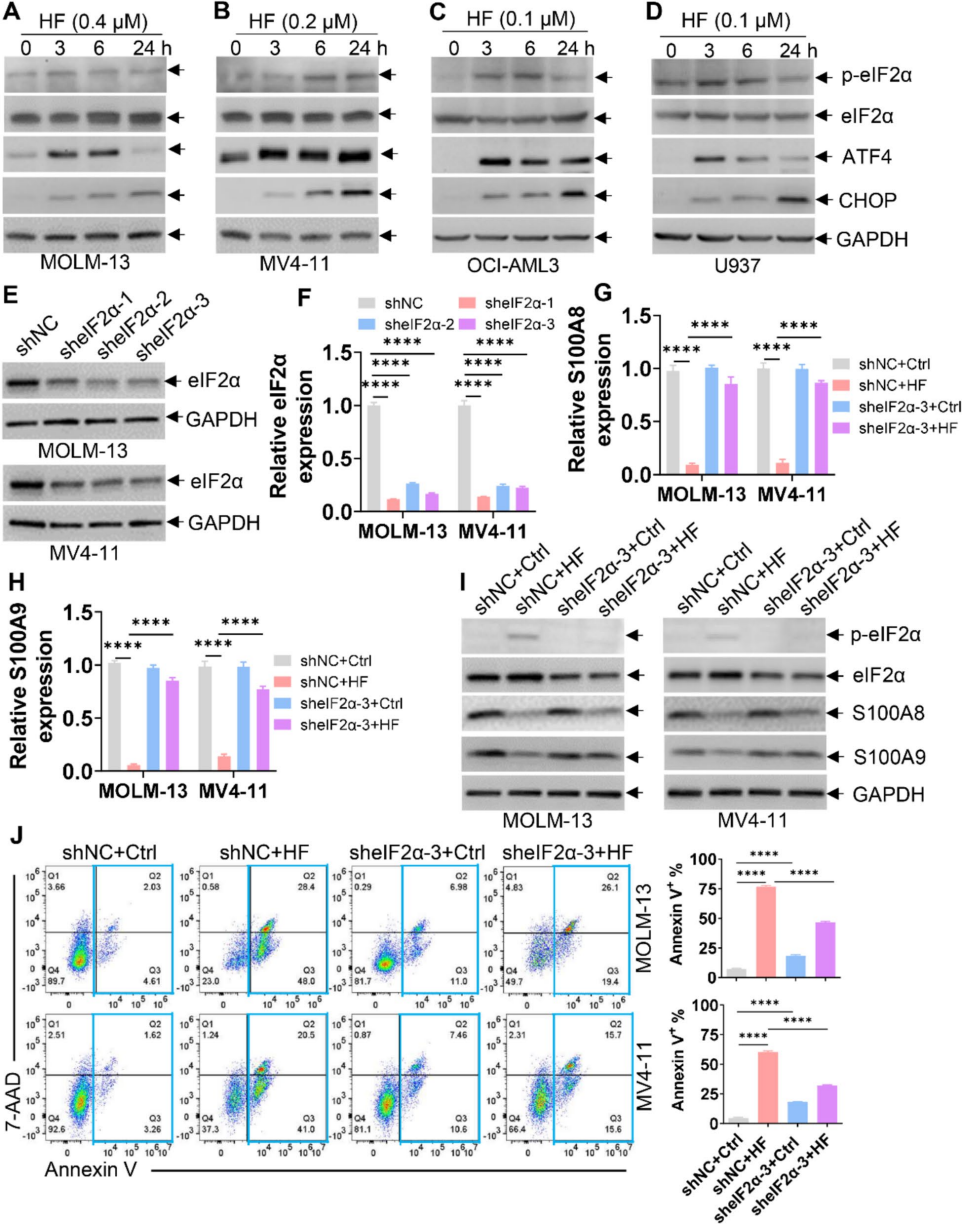
HF presents anti-leukemic effects by regulating p-eIF2α-S100A8/A9 signaling pathway. **A**–**D** The protein levels of ATF4, CHOP, eIF2α, and p-eIF2α^ser51^ were measured in four AML cell lines treated with HF for the indicated times. **E** and **F** The protein (**E**) and transcript (**F**) expressions of eIF2α were measured in MOLM-13 and MV4-11 cells transduced with shRNA for NC (shNC) or three shRNAs for eIF2α (sheIF2α) for 48 h, followed by puromycin (1 μg/mL) treatment for an additional 48 h. **G** and **H** The transcript expressions of S100A8 (**G**) and S100A9 (**H**) were measured in MOLM-13 and MV4-11 cells transduced with shNC or sheIF2α−3 and treated with or without HF (0.4 μM for MOLM-13; 0.2 μM for MV4-11) for 48 h. **I** The protein expressions of eIF2α, p-eIF2α^ser51^, S100A8, and S100A9 were measured in MOLM-13 and MV4-11 cells transduced with shNC or sheIF2α−3 and treated with or without HF (0.4 μM for MOLM-13; 0.2 μM for MV4-11) for 48 h. **J** Apoptosis was measured in MOLM-13 and MV4-11 cells transduced with shNC or sheIF2α−3 and treated with or without HF (0.4 μM for MOLM-13; 0.2 μM for MV4-11) for 48 h. Representative plots (left) and quantification of Annexin V^+^ cells (right) are shown. n = 3 or more independent biological replicates. ****P < 0.0001

Since HF activates AAR, characterized by the accumulation of p-eIF2α [10, 31], we therefore measured p-eIF2α protein expression in HF-treated and Ctrl cells. HF treatment markedly increased activating transcription factor 4 (ATF4), CHOP (encoded by DNA damage inducible transcript 3), and p-eIF2α protein levels compared with control treatment (Fig. 4A–D), confirming that HF activates AAR mediated by p-eIF2α-ATF4-CHOP signaling in AML cells. To investigate whether p-eIF2α mediates the downregulation of S100A8/A9 by HF, we depleted eIF2α expression using three different shRNAs, each of which efficiently reduced both mRNA and protein levels of eIF2α (Fig. 4E, F). AML cells with or without eIF2α knockdown were treated with or without HF. Knockdown of eIF2α prevented HF-induced decrease in S100A8/A9 transcript and protein expression (Fig. 4G–I). Furthermore, eIF2α knockdown partially rescued HF-induced apoptosis (Fig. 4J). Collectively, these results demonstrate that HF exerts anti-leukemic effects by regulating the eIF2α-S100A8/A9 signaling.

### HF treatment decreases global protein synthesis

**Fig. 5:**
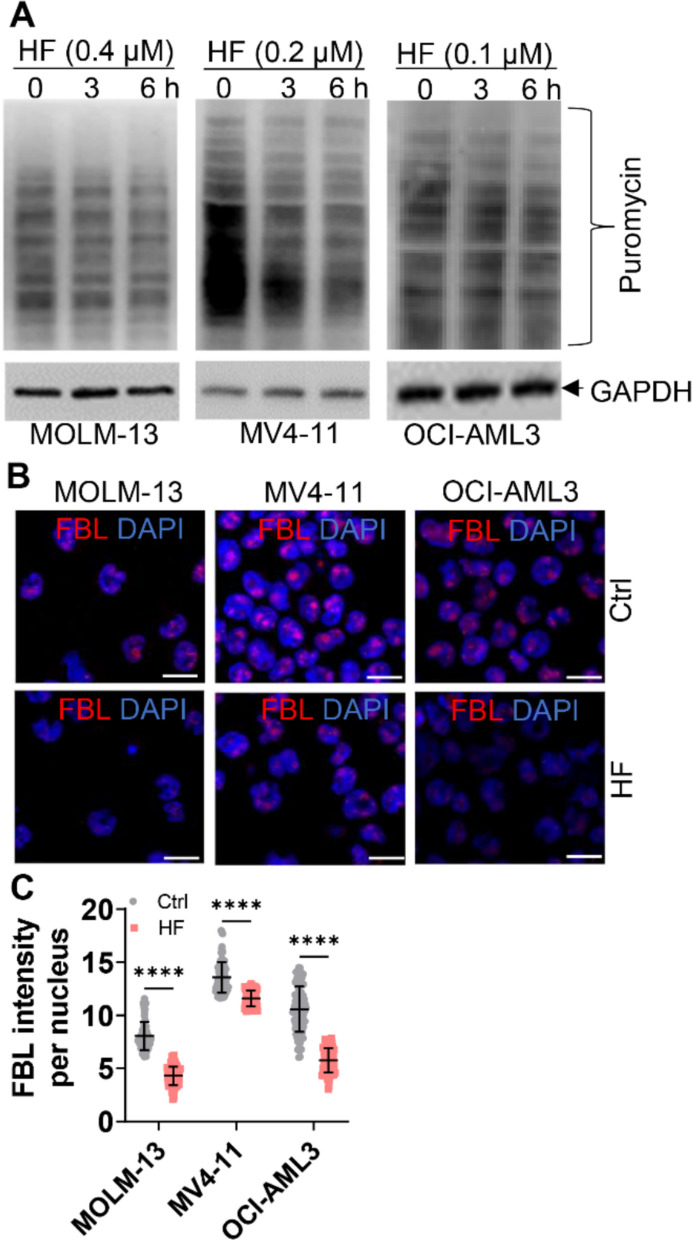
HF treatment inhibits global translation in AML cells. **A** AML cells were treated with indicated doses of HF for 3 and 6 h, followed by puromycin (10 μg/mL) incorporation for 30 min. Global protein synthesis was measured by surface sensing of translation (SUnSET) assay. **B** and **C** The mean fluorescence intensity (MFI) of nucleolar protein fibrillarin (FBL, red) was measured and quantified in AML cells treated with or without indicated doses of HF for 24 h. The representative immunofluorescence images (**B**) and quantitation of MFI per nucleus (**C**) are shown. Bar scales represent 10 μm. n = 3 or more independent biological replicates. ****P < 0.0001

Accumulation of p-eIF2α under AAR is known to inhibit global protein translation [33]. We therefore investigated the function of HF on translation inhibition. HF treatment caused a global decrease in protein synthesis by measuring the incorporation of puromycin in nascent peptides (Fig. 5A). We furthermore measured the expression of FBL, a key molecule in global protein synthesis and ribosome biogenesis [34]. We noticed a decreased IMF of FBL staining in HF-treated cells compared to untreated cells (Fig. 5B, C).

### Exogenous Pro rescues the deceased expression of S100A8/A9 and anti-leukemia ability of HF

**Fig. 6:**
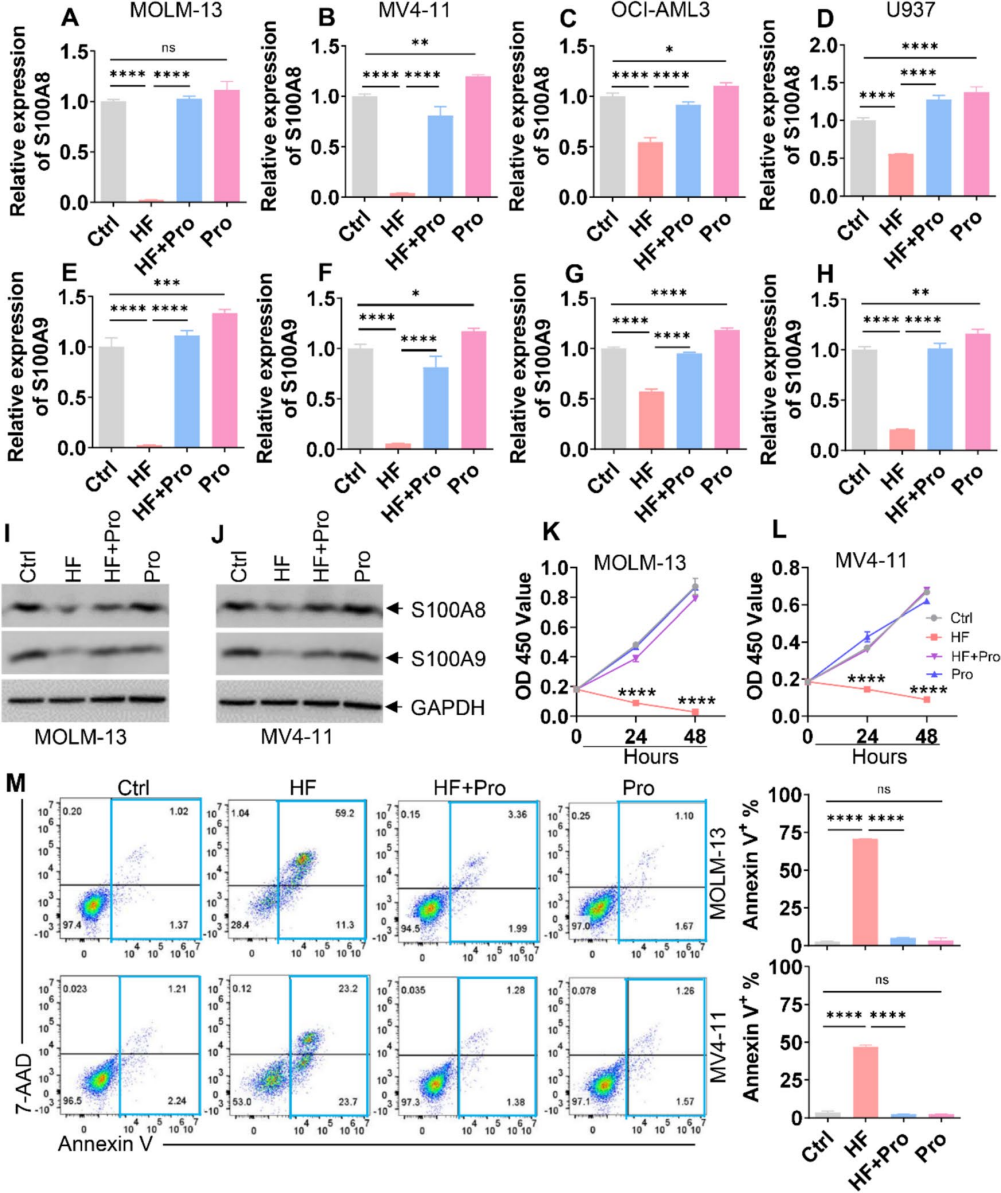
Exogenous proline (Pro) rescues the decreased expression of S100A8/A9 and the cytotoxic effects by HF. **A**–**H** The transcript expressions of *S100A8*/*A9* were measured in four AML cell lines treated with Control (Ctrl), HF (0.4 μM for MOLM-13; 0.2 μM for MV4-11; 0.1 μM for OCI-AML3; 0.1 μM for U937), HF + Pro (2 mM), or Pro for 24 h. **I** and **J** S100A8/A9 protein levels were measured in MOLM-13 and MV4-11 cells treated with Ctrl, HF (0.4 μM for MOLM-13; 0.2 μM for MV4-11), HF + Pro (2 mM), or Pro for 24 h. **K** and **L** Cell proliferation was assessed by CCK-8 assay in MOLM-13 and MV4-11 cells treated with Ctrl, HF (0.4 μM for MOLM-13; 0.2 μM for MV4-11), HF + Pro (2 mM), or Pro for 24 and 48 h. ****P < 0.0001 versus HF + Pro group. (**M**) Apoptosis was measured in MOLM-13 and MV4-11 cells treated with Ctrl, HF (0.4 μM for MOLM-13; 0.2 μM for MV4-11), Pro (2 mM), or HF + Pro for 24 and 48 h. Representative plots (left) and quantification of Annexin V^+^ cells (right) are shown. n = 3 or more independent biological replicates. *P < 0.05; **P < 0.01; ***P < 0.001; ****P < 0.0001. ns: not significant

Because HF inhibits prolyl-tRNA synthetase activity, and this inhibition can be blocked by the addition of exogenous Pro [9], we explored whether the addition of Pro could rescue HF-induced effects. The addition of Pro fully rescued the decreased mRNA expressions of S100A8/A9 in four AML cell lines treated with HF (Fig. 6A–H). Notably, Pro alone had minimal impact on expressions of *S100A8*/*A9* (Fig. 6A–H). Furthermore, the addition of Pro fully rescued HF-induced downregulation of S100A8/A9 protein levels in leukemic cells (Fig. 6I, J). Most importantly, exogenous Pro almost completely blocked HF-induced decrease in cell proliferation (Fig. 6K, L) and rescued HF-induced apoptosis (Fig. 6M) in MOLM-13 and MV4-11 cells. However, Pro alone did not affect proliferation (Fig. 6K, L) and apoptosis (Fig. 6M) in AML cells. These results confirm that the unavailability of Pro mediates HF-exerted anti-leukemic activity.

### HF enhances the cytotoxicity of chemotherapeutic drugs and reverses IDA resistance in AML cells

**Fig. 7:**
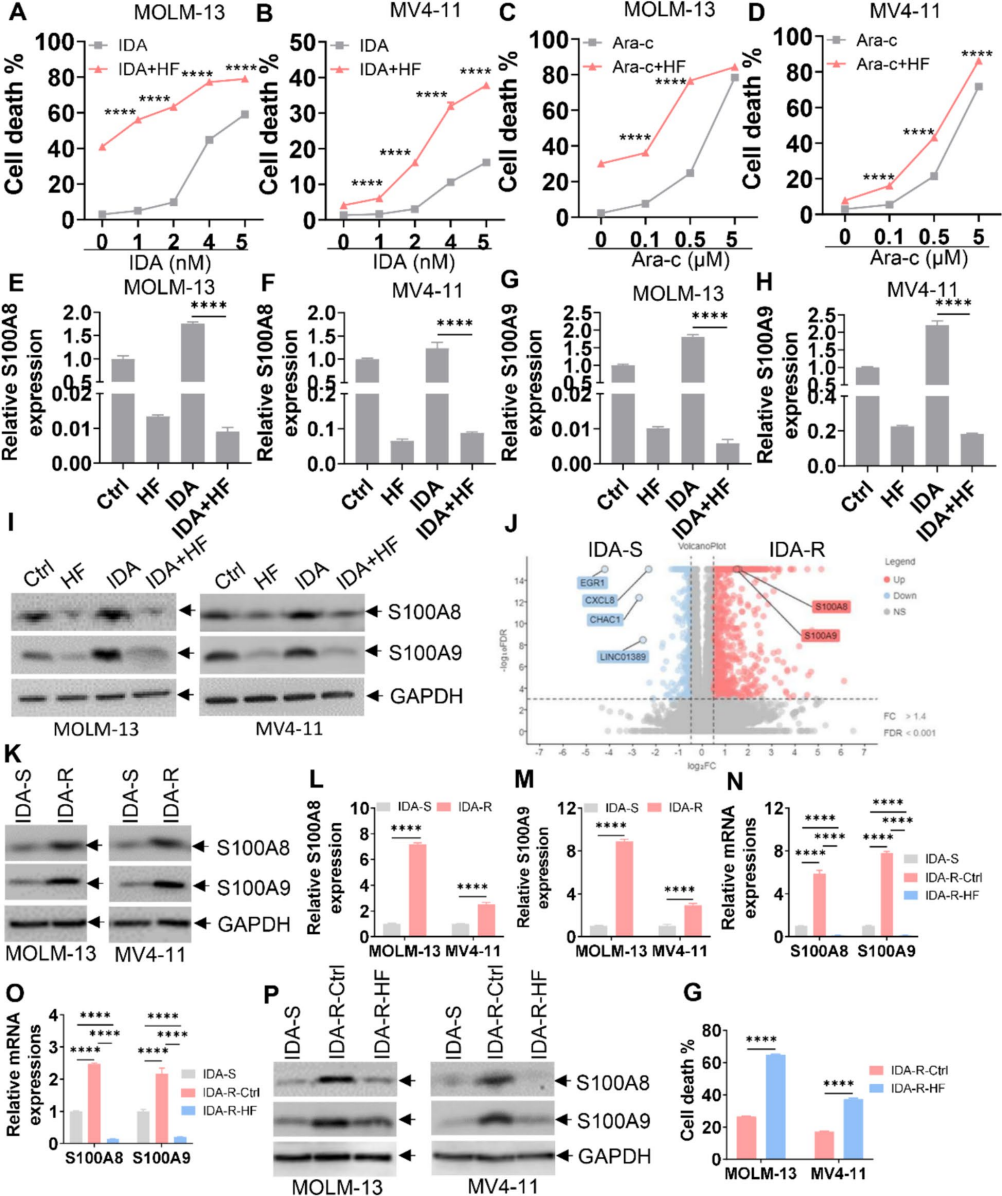
HF enhances the cytotoxicity of chemotherapeutic drugs and reverses idarubicin (IDA) resistance. **A** and **B** Cell death was measured by 7-AAD staining in MOLM-13 and MV4-11 cells treated with HF (0.2 μM for MOLM-13; 0.1 μM for MV4-11), various concentrations of IDA, and IDA + HF for 48 h. **C** and **D** Cell death was assessed by 7-AAD staining in MOLM-13 and MV4-11 cells treated with HF (0.2 μM for MOLM-13; 0.1 μM for MV4-11), different concentrations of cytarabine (Ara-C), and Ara-C + HF for 48 h. **E**–**H** Transcript expressions of *S100A8*/*A9* were measured in MOLM-13 and MV4-11 cells treated with HF (0.2 μM for MOLM-13; 0.1 μM for MV4-11), IDA (2 nM), and IDA + HF for 48 h. **I** Protein expressions of S100A8/A9 were measured in MOLM-13 and MV4-11 cells treated with HF (0.2 μM for MOLM-13; 0.1 μM for MV4-11), IDA (2 nM), and IDA + HF for 48 h. **J** RNA-seq analysis indicated upregulated or downregulated genes in MOLM-13-IDA-resistant (R) compared with MOLM-13-IDA-sensitive (S), cells. **K**–**M** The datasets used and/or analyzed during the current study are available from the corresponding author upon reasonable request.) The protein (**K**) and transcript (**L** and **M**) expressions of S100A8/A9 were measured in MOLM-13-IDA-(S), MOLM-13-IDA-(R), MV4-11-IDA-(S), and MV4-11-IDA-(R) cells. **N** Transcript expressions of S100A8/A9 were measured in MOLM-13-(S)-Ctrl, MOLM-13-(R)-Ctrl, and HF (0.2 μM)-treated MOLM-13-(R) cells for 24 h. **O** Transcript expressions of S100A8/A9 were measured in MV4-11-(S)-Ctrl, MV4-11-(R)-Ctrl, and HF (0.1 μM)-treated MV4-11-(R) cells for 24 h. **P** Protein expressions of S100A8/A9 were measured in MOLM-13-(S)-Ctrl, MOLM-13-(R)-Ctrl, HF (0.2 μM)-treated MOLM-13-(R) cells, MV4-11-(S)-Ctrl, MV4-11-(R)-Ctrl, and HF (0.1 μM)-treated MV4-11-(R) cells for 24 h. **G** Cell death was assessed by 7-AAD staining in MOLM-13-(R)-Ctrl, HF (0.4 μM)-treated MOLM-13-(R) cells for 48 h, MV4-11-(R)-Ctrl, and HF (0.2 μM)-treated MV4-11-(R) cells for 48 h. n = 3 or more independent biological replicates. ****P < 0.0001

Resistance to chemotherapeutic drugs is a major contributor to relapse and treatment failure in AML. To investigate whether HF can enhance drug cytotoxicity and reverse resistance, AML cells were treated with HF, idarubicin (IDA), cytarabine (Ara-C), hypomethylation agent decitabine (DAC), HDAC inhibitor tucidinostat (Tuc), or a combination of HF and these drugs. HF significantly increased cell death in AML cells treated with IDA or Ara-C (Fig. 7A–D). Similarly, HF enhanced Tuc (Fig. S10A, B) or DAC (Fig. S10C, D)-induced cell death compared with single treatment. We then investigated whether HF enhances IDA-induced cell death by regulating S100A8/A9. A low dose of IDA slightly increased the expression of S100A8/A9 (Fig. 7E–I); however, co-treatment with HF nearly abolished IDA-induced upregulation (Fig. 7E–I), suggesting that HF enhances IDA cytotoxicity by inhibiting S100A8/A9 expression.

To further assess whether HF could reverse IDA resistance, we generated MOLM-13 and MV4-11 cells resistant to IDA (IDA-R), which exhibited an approximately sevenfold higher IC50 for IDA compared with IDA-sensitive (IDA-S) cells (Fig. S11A, B). RNA-seq analysis revealed significantly higher S100A8/A9 expression in IDA-R cells compared with IDA-S cells (Fig. 7J), which was confirmed by Western blot (Fig. 7K) and qRT-PCR (Fig. 7L, M). Importantly, HF treatment markedly reduced S100A8/A9 transcript (Fig. 7N, O) and protein levels (Fig. 7P), as well as substantially induced cell death (Fig. 7G) in IDA-R cells.

To explore whether knockdown of *S100A8/A9* in IDA-R cells reduces IDA resistance, we measured the IC50 value for IDA in MOLM-13-R and MV4-11-R cells transduced with sh-NC or sh-S100A8/A9. Knockdown of *S100A8/A9* in MOLM-13-R and MV4-11-R cells significantly reduced the IC50 value compared with sh-NC cells (Fig. S12A, B), suggesting that S100A8/A9 deficiency reduces IDA resistance. Therefore, our results suggest that HF enhances the cytotoxicity of chemotherapeutic drugs and reverses IDA resistance by inhibiting S100A8/A9 expression.

### HF presents cytotoxic effects in *de no* AML samples and reverses IDA resistance in R/R AML samples

**Fig. 8:**
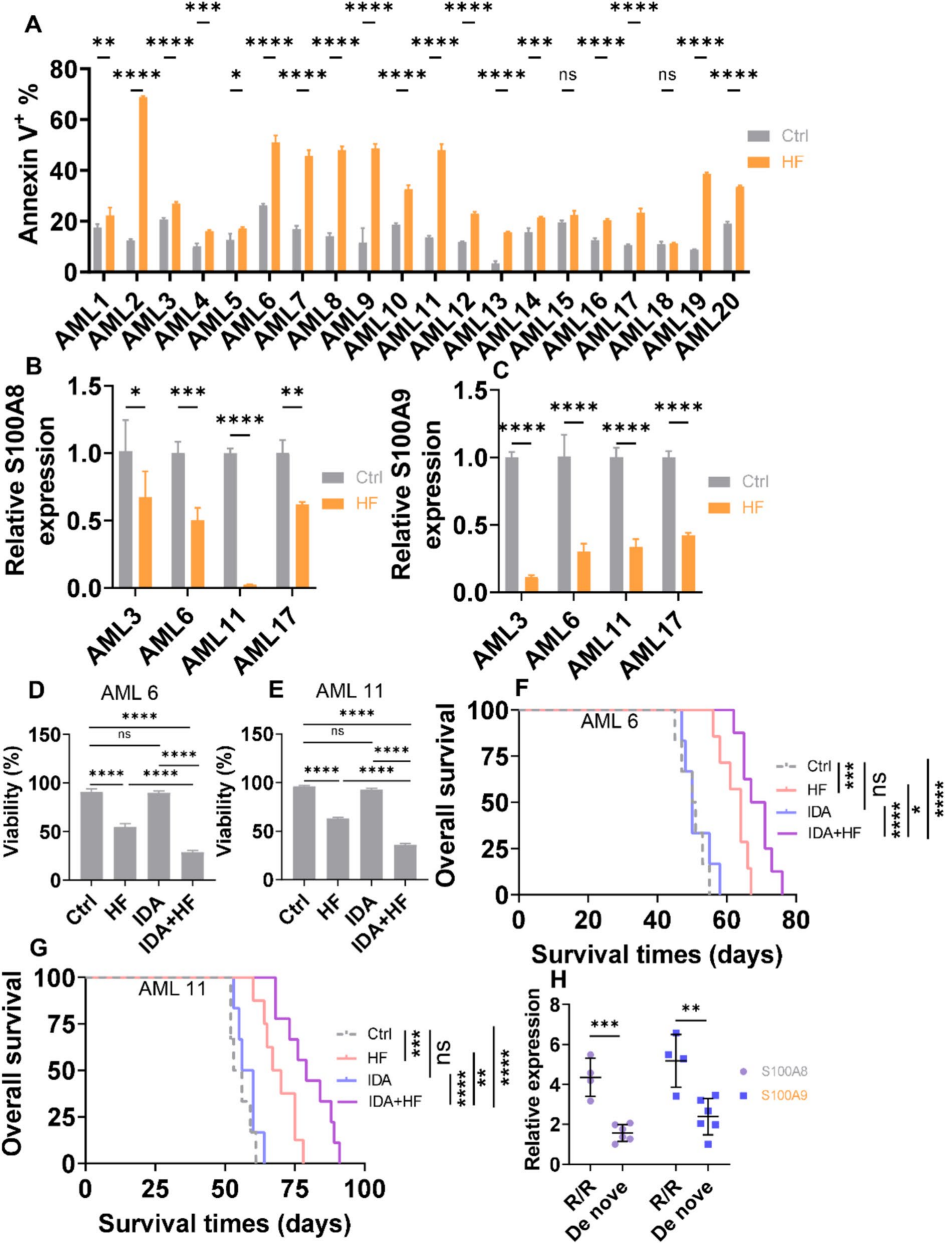
HF has cytotoxic effects and reverses IDA resistance in R/R AML samples. **A** Apoptosis was measured in BM mononuclear cells isolated from 20 AML patients and cultured with or without HF (0.1 μM) for 24 h. **B** and **C** S100A8/A9 mRNA expressions were measured in BM mononuclear cells isolated from 4 R/R AML patients and cultured with or without HF (0.1 μM) for 24 h. **D** and **E** Viability was measured in BM mononuclear cells from two R/R AML patients and cultured with Ctrl, HF (0.1 μM), IDA (2 nM), or their combination for 24 h. **F** and **G** OS was calculated in Ctrl (n = 6 for F; n = 6 for G), HF (n = 7 for F; n = 8 for G), IDA (n = 6 for F; n = 6 for G), and HF + IDA (n = 8 for F; n = 9 for G)-treated NSG mice xenografted with two R/R AML blasts. **H** S100A8/A9 mRNA expressions were measured in four R/R AML samples and six de novo AML samples. n = 3 or more independent biological replicates. *P < 0.05; **P < 0.01; ***P < 0.001; ***P < 0.0001; ****P < 0.0001. ns: not significant

To further explore the anti-leukemic effects of HF on AML cells, BM cells from 20 AML patients, including 16 de novo and 4 R/R AML patients (Table S3), were incubated with HF (0.1 μM) for 24 h, and apoptosis was measured. HF treatment induced apoptosis in 18 of the 20 AML samples (Figs. 8A, S13A–J, and S14A–J), and notably, all 4 R/R AML samples underwent significant apoptosis (Fig. 8A and Table S3). HF treatment also significantly reduced the transcript levels of *S100A8*/*A9* in all 4 R/R AML patients (Fig. 8B, C).

Given that HF enhances IDA-induced cell death and reverses IDA resistance in AML cell lines, we tested its effects in R/R AML patient samples. HF significantly restored IDA sensitivity in two R/R AML samples in vitro (Fig. 8D, E). Furthermore, the combination of HF and IDA substantially extended the OS compared with HF or IDA treatment alone in NSG mice xenografted with two R/R AML cells (Fig. 8F, G). Consistent with these findings, S100A8/A9 mRNA levels were higher in the four R/R AML samples compared with six de novo AML samples (Fig. 8H). Collectively, these results demonstrate that HF reverses IDA resistance in R/R AML patient samples.

### Anti-leukemia activity of HF in a murine leukemic model in vivo

**Fig. 9:**
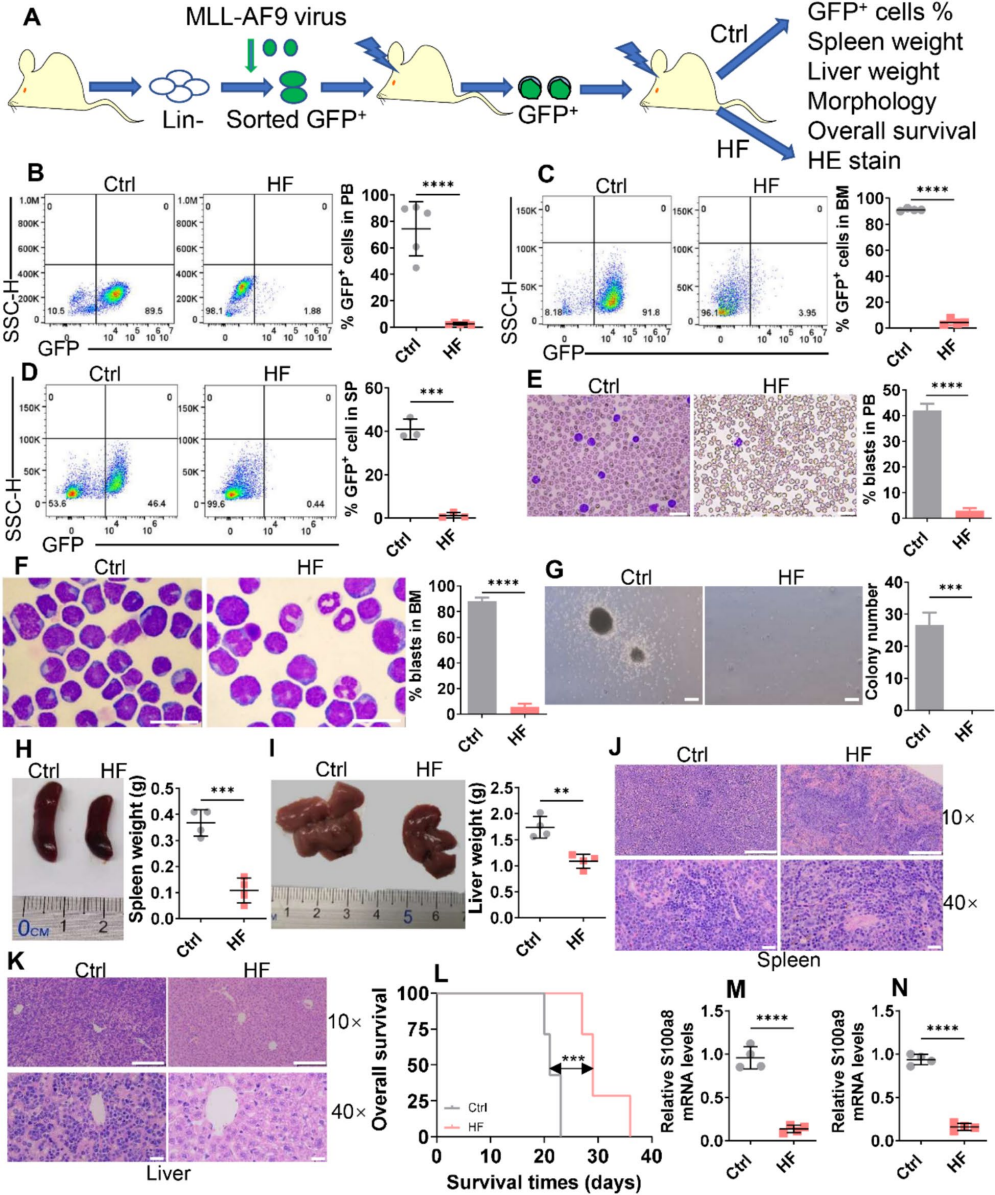
HF exerts anti-leukemic activity in the MLL-AF9-transformed murine AML model. **A** Outline of the MLL-AF9-induced murine AML model treated with Control (Ctrl) or HF. **B**–**D** The frequencies of GFP^+^ cells were measured in peripheral blood (PB, B), bone marrow (BM, C), and spleen (SP, D) mononuclear cells from Ctrl and HF-treated AML mice (n = 5 for PB, n = 4 for BM, and n = 3 for SP per group). Representative plots (left) and statistical analysis of GFP^+^ cells (right) are shown. **E** and **F** Wright-Giemsa staining was used to visualize PB (**E**) and BM (**F**) blasts from Ctrl and HF-treated AML mice. Representative images (left) and statistical analysis of the percentage of AML cells (right) are shown. Bar scales represent 20 μm for PB and BM. **G** Colony formation was counted in BM GFP^+^ cells isolated from Ctrl (n = 3) and HF (n = 3)-treated leukemic mice. Bar scales represent 20 μm. **H** and **I** Spleen and liver tissues were isolated from Ctrl and HF-treated AML mice (n = 4 for spleen and liver from each group), and their weights were calculated. Representative images (left) and statistical analysis of spleen and liver weights (right) are shown. **J** and **K** Representative images of HE staining of spleen and liver tissues from Ctrl and HF-treated leukemic mice. Bar scales represent 200 μm (10 ×) and 20 μm (40 ×) for spleen and liver tissues. (**L**) Overall survival was calculated in Ctrl (n = 7) and HF (n = 7)-treated leukemic mice. (**M** and **N**) Transcript expressions of *S100a8* and *S100a9* were measured in BM GFP^+^ cells from Ctrl (n = 4) and HF (n = 4)-treated leukemic mice. **P < 0.01; ***P < 0.001; ***P < 0.0001

To further evaluate the anti-leukemic effects of HF in vivo, we employed an MLL-AF9-transformed murine AML model. Murine GFP^+^ AML cells were xenografted into recipient mice, which were treated with HF or vehicle control (Ctrl) (Fig. 9A). GFP^+^ cells representing leukemic burden in PB, BM, and spleen were first measured. HF treatment substantially reduced the percentage of leukemic cells in PB (Fig. 9B), BM (Fig. 9C), and spleen (Fig. 9D). Wright-Giemsa staining revealed a marked reduction in leukemic blasts in both PB (Fig. 9E) and BM (Fig. 9F) of HF-treated mice compared with Ctrl mice. Furthermore, BM GFP^+^ cells were isolated from HF-treated mice and Ctrl mice for colony formation. HF treatment almost completely inhibited colony formation (Fig. 9G). The spleen and liver weights were also measured to assess the effects of HF on leukemic cell infiltration. HF treatment reduced spleen weight by about 70% (Fig. 9H) and liver weight by approximately 60% (Fig. 9I) compared with the Ctrl group. Furthermore, HF treatment substantially eradicated the infiltration of AML cells in spleen and liver tissues by HE staining (Fig. 9J, K). OS was higher in the HF-treated mice than in the Ctrl mice (Fig. 9L). In addition, HF treatment significantly decreased the *S100a8* and *S100a9* mRNA levels in BM GFP^+^ cells (Fig. 9M, N).

### HF has little toxic effect on normal hematopoiesis

We next investigated the potential toxic effects of HF on normal hematopoiesis. We first conducted a cell count in the PB of HF-treated and Ctrl mice one month after the last HF injection. HF treatment did not significantly affect white blood cell (WBC), red blood cell (RBC), hemoglobin (HGB), and platelet (PLT) counts (Fig. S15A–D). Furthermore, HF treatment did not alter spleen and liver weight (Fig. S15E, F). Colony formation was performed using BM c-Kit^+^ cells isolated from wild-type mice treated with or without HF. HF treatment did not significantly affect colony numbers (Fig. S15G, H). Also, the CFU-M, CFU-GM, CFU-GEMM, BFU-E, and BFU-E colonies were equal in HF-treated cells than in Ctrl cells (Fig. S15I). These results suggest that HF does not adversely impact normal hematopoiesis. Furthermore, HF treatment did not affect the structure of liver, spleen, kidney, and intestine (Fig. S15J), indicating that HF has minimal toxic effects in vivo.

## Discussion

**Fig. 10:**
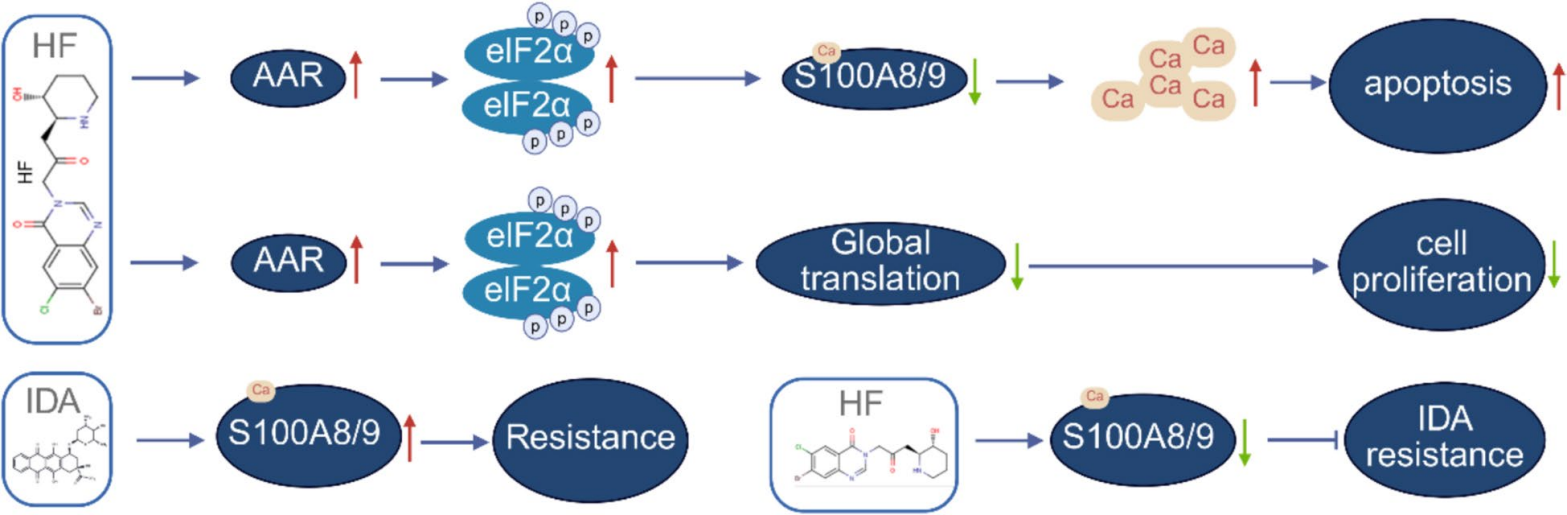
Schematic diagram of the broad-spectrum anti-leukemic ability of HF. HF activates AAR, leading to the accumulation of p-eIF2α, which inhibits S100A8/A9 transcript and protein levels, followed by the upregulation of cytoplasmic Ca^2+^ level. High level of cytoplasmic Ca^2+^ triggers apoptosis. Thus, HF induces apoptosis by regulating p-eIF2α-S100A8/A9-Ca^2+^ signaling. HF activated p-eIF2α, followed by downregulation of global protein synthesis and global translation. Low protein synthesis inhibits proliferation. Therefore, HF inhibits proliferation by regulating p-eIF2α-global translation. The increased expression of S100A8/A9 is a marker of IDA resistance. HF reverses IDA resistance by inhibiting S100A8/A9

In this study, we comprehensively investigated the broad-spectrum cytotoxic effects of HF on AML cells. HF-induced AAR leads to phosphorylation of eIF2α, which decreases S100A8/A9 expression, resulting in increased cytoplasmic Ca^2^⁺ levels. Elevated Ca^2+^ facilitates apoptosis, indicating that HF induces cell death via the p-eIF2α-S100A8/A9-Ca^2^⁺ signaling axis (Fig. 10). HF-induced p-eIF2α inhibits global protein synthesis, reduces translation, and suppresses proliferation. Accordingly, HF inhibits proliferation by regulating p-eIF2α-global translation (Fig. 10). In addition, HF reverses IDA resistance by blocking IDA-induced S100A8/A9 upregulation (Fig. 10). Importantly, HF exerts anti-leukemic activity in de novo AML patients and reverses IDA resistance in R/R AML patients, while sparing normal hematopoiesis. In conclusion, our findings highlight HF as a potential therapeutic strategy for AML, particularly R/R patients.

The accumulation of p-eIF2α is a hallmark of AAR or cellular stress in eukaryotic cells, which inhibits eIF2α activity and reduces translation initiation [35, 36]. Our results show that HF enhances p-eIF2α levels in AML cells, and this activation of p-eIF2α mediates the anti-leukemia activity of HF, underscoring p-eIF2α as a critical mediator of HF activity. These results are consistent with the report that all-trans retinoic acid and arsenic trioxide induce p-eIF2α expression, leading to the reduction of oncoproteins required for proliferation and survival in acute promyelocytic cells [37]. Furthermore, AML patients with lower levels of p-eIF2α exhibit higher frequency of relapse compared with those with higher p-eIF2α [37], further supporting the notion that triggering p-eIF2α expression may have therapeutic benefit.

S100A8/A9 can regulate multiple signaling pathways, including intracellular calcium, oxidative stress, and apoptosis in tumor cells [38]. Previous reports have highlighted S100A8/A9 as potential therapeutic targets in AML [21, 30]. For instance, high expression of S100A9 in AML is associated with altered mitochondrial metabolism and sensitivity to venetoclax [30]. However, the regulation of S100A8/A9 in the context of AAR remains unexplored. Our results demonstrate that HF-and EBSS-induced AAR substantially inhibits S100A8/A9 transcript and protein levels. Furthermore, the Pro supplement completely rescues the decreased S100A8/A9 expression induced by HF, indicating that HF-induced Pro unavailability triggers AAR, which in turn inhibit S100A8/A9 expression. Although HF-and EBSS-induced AAR typically inhibit translation initiation [35], our results suggest that AAR can still regulate transcript levels of specific genes essential for leukemic survival, consistent with prior reports showing that EPRS inhibitor-induced AAR affects transcripts involved in ribosomal processes and RNA binding [14]. Thus, HF exerts its cytotoxic effects through AAR-mediated p-eIF2α-S100A8/A9 signaling in AML.

Rapidly proliferating AML cells require high rates of protein synthesis to support growth [39]. Translation inhibitors, chemotherapeutics, and UV radiation inhibit global translation by impairing ribosome function [33, 40], which might facilitate the unwanted side effects of genotoxic cancer therapies. However, HF treatment inhibits global protein synthesis by triggering AAR, a physiological response to nutrient deprivation, suggesting a lower risk of adverse effects. Furthermore, homeostatic HSCs maintain low global translation rates to preserve energy for long-term maintenance [41], suggesting that HF might have low toxic effects on the HSC population, which is consistent with our results.

Drug resistance and relapse represent significant challenges in the treatment of AML patients [42]. Here, we unexpectedly found that HF enhances the cytotoxicity of several chemotherapeutic drugs, including IDA, Ara-C, DAC, and Tuc. Notably, HF facilitates IDA-induced cytotoxicity by inhibiting IDA-induced upregulation of S100A8/A9 in AML cells. These results are consistent with reports showing that elevated levels of S100A8/A9 correlate with resistance to gilteritinib [23] and prednisolone [22]. Therefore, S100A8/A9 inhibition may reduce resistance to chemotherapeutic drugs, highlighting S100A8/A9 as critical mediators of chemoresistance. Consistent with this, knockdown of S100A8/A9 reverses IDA resistance in AML cells. Interestingly, low-dose IDA treatment slightly increases S100A8/A9 expression, whereas IDA-R cells exhibit substantially higher levels than IDA-S cells, suggesting that prolonged IDA exposure may trigger S100A8/A9 upregulation. However, the molecular mechanisms underlying this induction remain to be elucidated. Significantly, HF effectively reverses IDA resistance in IDA-R cells in vitro and reverses IDA resistance in R/R AML patient samples-xenografted NSG mice in vivo, supporting the potential preclinical utility of HF for treating R/R AML.

S100A8/A9 heterodimers bind cytoplasmic Ca^2+^ to form heterotetrameric protein complexes [43], which can regulate apoptosis, differentiation, and cell proliferation [38, 44]. In this study, HF treatment decreases S100A8/A9 expression, while knockdown of S100A8/A9 increases cytoplasmic Ca^2+^ levels. Therefore, HF triggers cytoplasmic Ca^2+^ levels by decreasing S100A8/A9 expression. As an important second messenger, elevated cytoplasmic Ca^2+^ levels promote apoptosis by regulating mitochondrial dysfunction [45, 46] or endoplasmic reticulum stress [47, 48]. Our results demonstrate that cytoplasmic Ca^2+^ plays a key role in mediating HF-induced cytotoxic effects. This is consistent with the report showing that prednisolone triggers apoptosis by elevating Ca^2+^ levels via S100A8/A9 downregulation [22].

Although HF exhibits broad-spectrum cytotoxic effects in leukemic cells by regulating p-eIF2α-S100A8/A9-Ca^2+^ signaling, the definite molecular mechanism by which p-eIF2α mediates HF-induced downregulation of S100A8/A9 requires further investigation. Similarly, the mechanism underlying the induction of S100A8/A9 following prolonged IDA treatment remains unclear.

In conclusion, HF, at a relatively low concentration, exerts broad-spectrum cytotoxic effects through activating AAR-mediated p-eIF2α-S100A8/A9-Ca^2+^ signaling and inhibiting global protein synthesis. These findings position HF as a promising therapeutic candidate for AML, particularly those with R/R AML.

## Supplementary Information

Supplementary Material 1. 

## Data Availability

The datasets used and/or analyzed during the current study are available from the corresponding author upon reasonable request.

## Abbreviations

AML: Acute leukemia myeloid
AAR: Activation of amino acid starvation response
Ara-C: Cytarabine
APL: Acute promyelocytic leukemia
BMT: Bone marrow transplantation
DAC: Decitabine
EPRS: Glutamyl-prolyl-tRNA synthetase
eIF2α: Eukaryotic translation initiation factor 2α
H&E: Hematoxylin and eosin
HSPCs: Hematopoietic stem progenitor cells
HF: Halofuginone
IDA: Idarubicin
NSG: NOD/SCID‑IL2Rγ
qRT‒PCR: Quantitative RT‒PCR
OS: Overall survival
R/R-AML: Relapsed and refractory AML
Tuc: Tucidinostat
MMP: Mitochondrial membrane potential
