# Supplementary material for: Halofuginone exerts broad-spectrum cytotoxic effects by regulating p-eIF2α-S100A8/A9-calcium signaling, inhibiting global protein synthesis, and reversing the resistance of idarubicin in acute myeloid leukemia
Source: Chin Med. 2026 Jan 6;21:7. doi: 10.1186/s13020-025-01278-9 (PMC12771945; doi:10.1186/s13020-025-01278-9)
Supplement: Supplementary file 1 — Supplementary Material 1. [file 13020_2025_1278_MOESM1_ESM.docx]

**Supplemental Methods**

**Reagents**

HF (TargetMol, Boston, MA, USA), puromycin (Sigma–Aldrich, St. Louis, MO, USA), idarubicin (IDA, TargetMol), ethylene glycol tetraacetic acid (EGTA, TargetMol), cytarabine (Ara-C, TargetMol), decitabine (DAC, TargetMol), BAPTA-AM (TargetMol), and tucidinostat (Tuc, TargetMol) were dissolved in DMSO and deposited at -86 °C until use.

**Apoptosis assay**

Apoptosis was measured using annexin V/7-AAD staining (BD PharMingen, San Diego, CA, USA). Briefly, leukemic cells (2×10^5^ cells/mL) were collected from six-well plates and washed with 1×binding buffer. Cells were incubated in 100 μL 1×binding buffer containing 5.0 μL annexin V-APC and 5.0 μL 7-AAD for 30 min at room temperature in the dark. After incubation, AML cells were finally resuspended in 400 μL of 1×binding buffer. Apoptotic cells were analyzed by flow cytometry (CytoFLEX LX, Beckman-Coulter, Brea, CA, USA) within 30 min after staining. Apoptotic cells were calculated as Annexin V^+^/7-AAD^+^ (late apoptosis) + Annexin V^+^/7-AAD^-^ (early apoptosis).

**Mitochondrial membrane potential (MMP) assay**

The fluorescent carbocyanine dye (JC-1) labeling of mitochondria was performed to assess MMP. JC-1 probe and 1×buffer (Beyotime Biotechnology, Nanjing, Jiangsu, China) were mixed to form the JC-1 staining buffer. AML cells (about 5×10^5^/sample) were collected, washed with phosphate-buffered saline (PBS), and incubated with JC-1 staining buffer for 20 min at 37 °C in a humidified incubator with 5% CO_2_. JC-1 with red (490ex/530em nm) representing MMP was measured by flow cytometry (CytoFLEX LX, Beckman-Coulter).

**Colony-forming unit assay**

Murine GFP^+^ leukemic cells were sorted from MLL-AF9-transduced leukemic mice. Murine c-Kit^+^ cells were sorted from wild-type C57/B6 mice. Both murine GFP^+^ leukemic and normal c-Kit^+^ cells were plated into murine methylcellulose medium (MethoCult™ GF M3434, Stemcell Technologies, Vancouver, BC, Canada) for 10 days to count colonies. Human leukemic cells were seeded into human methylcellulose medium (MethoCult^TM^ H4434 Classic, Stemcell Technologies), and colonies (> 40 cells) were counted 10 days after plating according to the manufacturer's protocol.

**Cell cycle analysis**

Leukemic cells (2×10^6^) were collected, washed with 1×PBS buffer, fixed with 70% ethanol, and pre-treated with 10 μg/mL of RNase (Beyotime Biotechnology). AML cells were then incubated for 30 min at room temperature in 1×PBS buffer containing propidium iodide (0.04 mg/mL, Beyotime Biotechnology). Cells were acquired by flow cytometry (Beckman-Coulter, Brea, CA, USA), and FlowJo software v10.0 (Ashland) was used to assess the distribution of cells in the G0/G1, G2/M, and S phases.

**Cell proliferation by CCK-8 assay**

Leukemic cells (100 μL, 1×10^5^ cells/mL) were seeded in 96-well plates. Then, CCK-8 solution (10 μL, TargetMol) was added and incubated for 2–4 h. The absorbance was measured at 450 nm using an MRX II microplate reader (Dynex, Chantilly, VA, USA).

**Viability assay in primary AML cells**

Cell viability was measured by the trypan blue exclusion test. Leukemic cells were mixed with trypan blue (0.1%, Thermo Scientific, Waltham, MA, USA), and cell viability was measured by a cell counter (VI-CELL XR, Beckman-Coulter).

**Western blot**

Western blot analysis was performed according to the standard procedure. Briefly, total protein lysates were extracted by 1×RIPA buffer with protease and phosphatase inhibitors (Thermo Scientific) after AML samples were collected and washed twice with ice-cold PBS. Cell lysates were incubated on ice for 30 min, followed by centrifugation at 12,000×g for 15 min to obtain supernatants. Protein concentration was measured using a BCA assay (Thermo Scientific). Equal protein amounts (40 μg/sample) were loaded and separated by 8-15% SDS-PAGE, then transferred to 0.22 μM polyvinylidene fluoride membranes (PVDF, Bio-Rad, Richmond, CA, USA). Membranes were incubated with primary antibodies at 4 ℃ overnight after they were blocked with 5% non-fat milk at room temperature for 2 h. Furthermore, membranes were incubated with horseradish peroxidase-conjugated secondary antibodies after washing five times by 1×TBST. Signals were detected using chemiluminescence reagents (Thermo Scientific). The following antibodies were used: S100A8 (66853-1-Ig, Proteintech, Wuhan, Hubei, China); S100A9 (26992-1-AP, Proteintech, Wuhan, Hubei, China); ATF4 ([ab1371](https://www.abcam.cn/products/primary-antibodies/atf-4-antibody-c-terminal-ab1371), Abcam, Cambridge, MA, USA), CHOP (2895, Cell Signaling Technology, Beverly, MA, USA), eIF2α (5324, Cell Signaling Technology); p-eIF2α^ser51^ (9721, Cell Signaling Technology); cleaved poly (ADP-ribose) polymerase (PARP, #5625, Cell Signaling Technology); cleaved caspase-3 (#9664, Cell Signaling Technology); puromycin (1:5000, MABE343, Millipore, Billerica, MA, USA). GAPDH (ab8245, Abcam) or β-actin (ab6276, Abcam) antibody was used as an endogenous control.

**Cytoplasmic Ca^2+^ level assay**

Detection of cytoplasmic Ca^2+^ was performed according to the manufacturer's instructions (Dojin Laboratories, Kumamoto, Japan). Briefly, 2×10^5^ AML cells were resuspended in 200 µL of 1×Hank's balanced salt solution. Fluo-4 Ca^2+^ assay buffer (200 µL) was added to the cell suspension. The mixture was incubated at 37 °C for 30 min. Fluo-4 signals were collected and analyzed by flow cytometer (CytoFLEX LX, Beckman-Coulter). Ca^2+^ inhibitors EDTA and BAPTA-AM were added as controls to explore the mechanism of action of HF.

**Hematoxylin and eosin (H&E) stain**

Spleen and liver tissues from mice were fixed in formalin and embedded in paraffin. The tissue samples were dehydrated and stained with H&E following standard protocols.

**Wright-Giemsa stain**

For morphological analysis, murine BM cytospins and PB smears were stained with Wright-Giemsa stain according to standard protocols[1]. Briefly, BM mononuclear cells (2×10^5^/mL) suspended in PBS (100 μL) were collected and centrifugated onto slides. Murine PB (2–3 μL) was placed on slides. After the slides were air-dried, Wright-Giemsa staining buffer (1.0 mL) was applied for 3–4 min at room temperature. BM cytospins and PB smears were observed under an optical microscope (DM 2000 LED, Leica Microsystems Inc., Deerfield, IL, USA) after the slides were washed and thoroughly dried.

**Immunological fluorescence (****IMF) staining**

AML cells were collected on slides and centrifuged by a cytospin (Shandon, Runcorn, United Kingdom). After slides were wholly dried, cells were fixed and permeabilized with 0.1% (v/v) Triton X-100 in 1×PBS solution. AML cells were incubated with anti-fibrillarin (FBL) antibody (1:800) at 4 ℃ overnight after cells were blocked with 2% (w/v) BSA in 1×PBS buffer. Then, cells were stained with secondary antibody conjugated with Texas-Red (1:400, Beyotime Biotechnology) for 1 h at room temperature. Finally, cellular DNA was stained with 4,6-diamidino-2-phenylindole (DAPI; Beyotime Biotechnology). Fluorescence signals were detected by a laser scanning confocal microscope (Stellaris 5, Leica Microsystems Inc.) and quantified by Image J software (NIH, USA).

**Construction of IDA-resistant cells**

AML cells were cultured with gradually increasing concentrations of IDA from 0.1 nM to 12.8 nM. At approximately 3 weeks, the IDA dose was gradually increased by 2-fold in the culture. AML cells were collected every other day and centrifuged to remove the supernatant. IDA was added to the lipid supernatant during the construction of IDA-resistant cells. The half-maximal inhibitory concentration (IC50) was measured in both IDA-resistant and sensitive cells to confirm the successful construction of the IDA-resistant cell line.

**RNA sequencing (RNA-seq) analysis**

MOLM-13 cells were treated with HF (0.2 μM) for 24 h. Total mRNA from HF-treated and untreated MOLM-13 cells was extracted using TRIzol reagent (Invitrogen). mRNA was fragmented into small pieces using the fragmentation buffer. A cDNA library was constructed from the suitable fragments via PCR amplification. Sequencing was performed on the Illumina HiSeq 4000 platform to obtain image analysis, base calling, and error estimation by Illumina/Solexa Pipeline. StringTie (version 1.2.3) was used to produce the transcriptome after the trimmed reads were mapped to the corresponding reference genome by HISAT2 (version 2.0.4). The software Integrative Genomics Viewer and the R packages were performed for data visualization and analysis of differentially expressed genes (DEGs). DEG between Control (Ctrl) and HF-treated samples were identified if they had an adjusted *P*-value lower than 0.05 and a fold change of more than 2.0. Pathway enrichment analyses were conducted using an online curated gene set collection.

**Enrolled AML patients**

We conducted a retrospective cohort study to review the untreated AML patients who receives standard induction chemotherapy between January 2020 and December 2024 in the First Affiliated Hospital of Wenzhou Medical University. AML patients were diagnosed and classified according to the French–American–British (FAB) and the 2016 World Health Organization (WHO) criteria[2, 3]. Patients with acute promyelocytic leukemia (APL) was excluded in this study. 94 AML patients were treated with standard chemotherapy. The standard induction regimen for AML consists of continuous intravenous infusion of cytarabine at a dose of 100-200 mg/m²/day for 7 days, concurrently with an anthracycline, idarubicin administered at a dose of 12 mg/m²/day via intravenous push for the first 3 days. Complete remission (CR) was defined as the absence of detectable leukemia cells in the bone marrow (<5% blasts), absence of circulating blasts and extramedullary disease, and recovery of normal blood counts. The transcript expression of S100A8/A9 was measured in BM cells from 94 AML patients by qRT-PCR. AML patients with higher or lower S100A8/A9 expression were determined by median level. Our research was approved by the Institutional Ethics Committee of the First Affiliated Hospital of Wenzhou Medical University, as well as the *Helsinki Declaration* of 1975, as revised in 2013. All AML patients have provided informed consent for their participation.

References

1. Song MG, Gao SM, Du KM, Xu M, Yu Y, Zhou YH, et al. Nanomolar concentration of NSC606985, a camptothecin analog, induces leukemic-cell apoptosis through protein kinase Cdelta-dependent mechanisms. Blood*.* 2005;105(9):3714-3721.

2. Arber DA, Orazi A, Hasserjian R, Thiele J, Borowitz MJ, Le Beau MM, et al. The 2016 revision to the World Health Organization classification of myeloid neoplasms and acute leukemia. Blood*.* 2016;127(20):2391-2405.

3. Bennett JM, Catovsky D, Daniel MT, Flandrin G, Galton DA, Gralnick HR, et al. Proposed revised criteria for the classification of acute myeloid leukemia. A report of the French-American-British Cooperative Group. Ann Intern Med*.* 1985;103(4):620-625.

**Supplemental figures and legends**

**
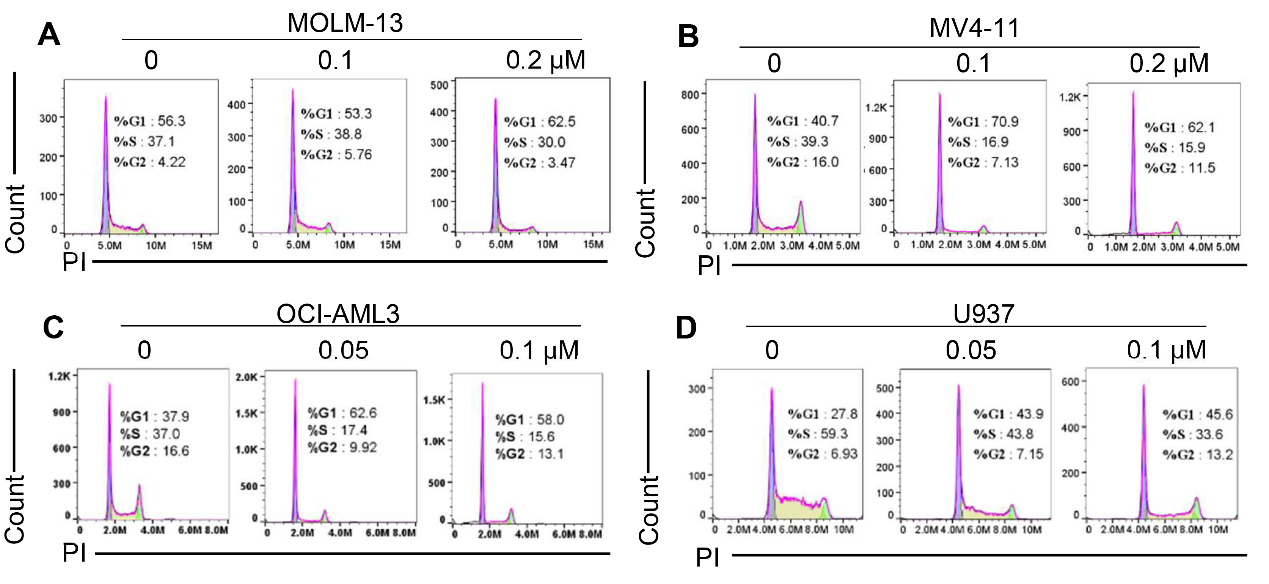
**

**Figure S1: HF induces cell cycle arrest in AML cell lines.** (A–D) Cell cycle distribution was measured by PI staining in four AML cell lines treated with different concentrations of HF for 24 h. The representative cell cycle distribution plots are shown.

**
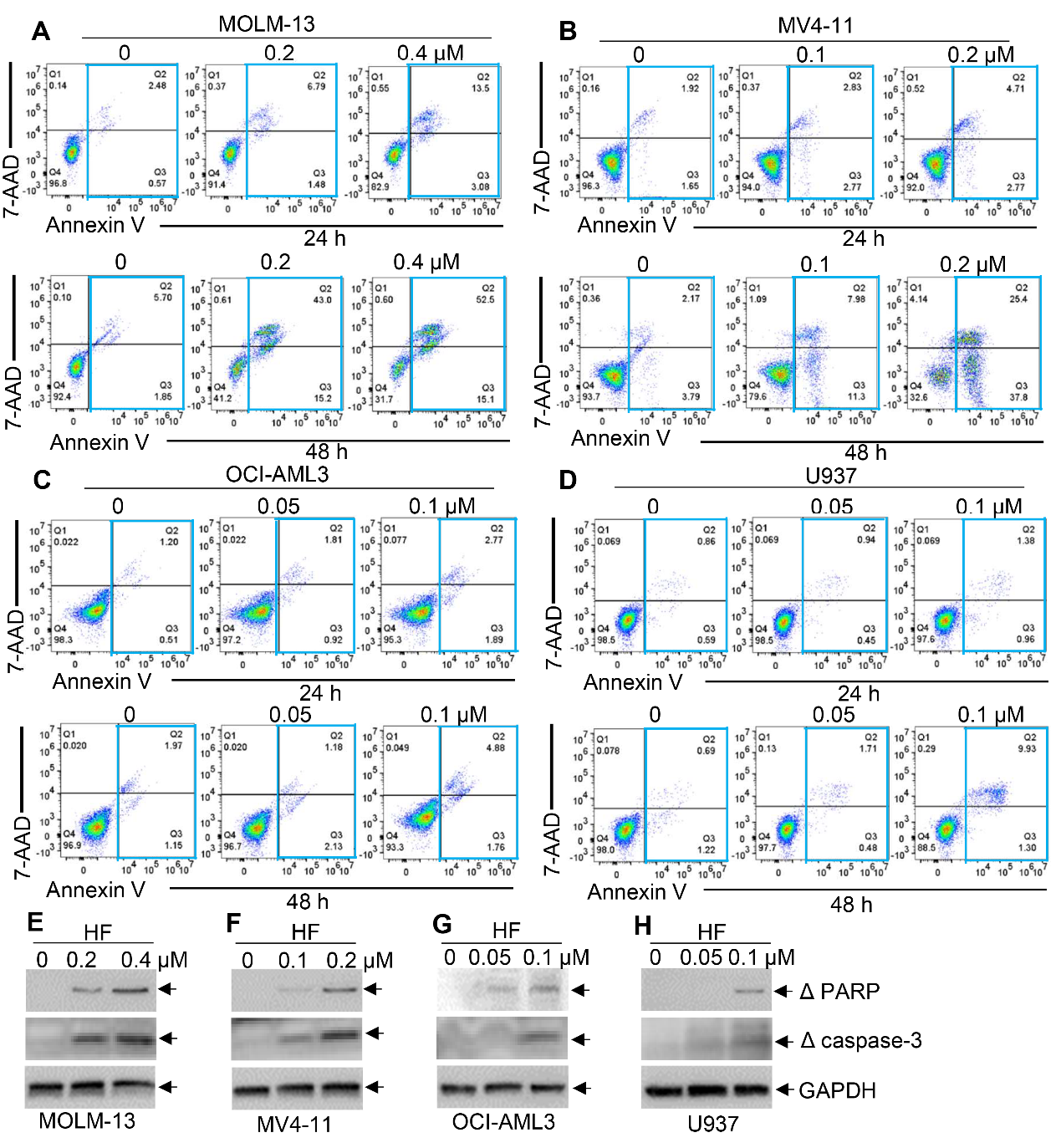
**

**Figure S2: HF induces apoptosis in AML cell lines.** (A–D) Apoptosis was measured by Annexin V/7-AAD staining in four AML cell lines treated with the indicated concentrations of HF for 24 and 48 h. The representative plots of Annexin V/7-AAD staining are shown. (E–H) The protein expression levels of cleaved PARP (ΔPARP) and caspase-3 (Δcaspase-3) were measured in four AML cell lines treated with indicated concentrations of HF for 48 h.


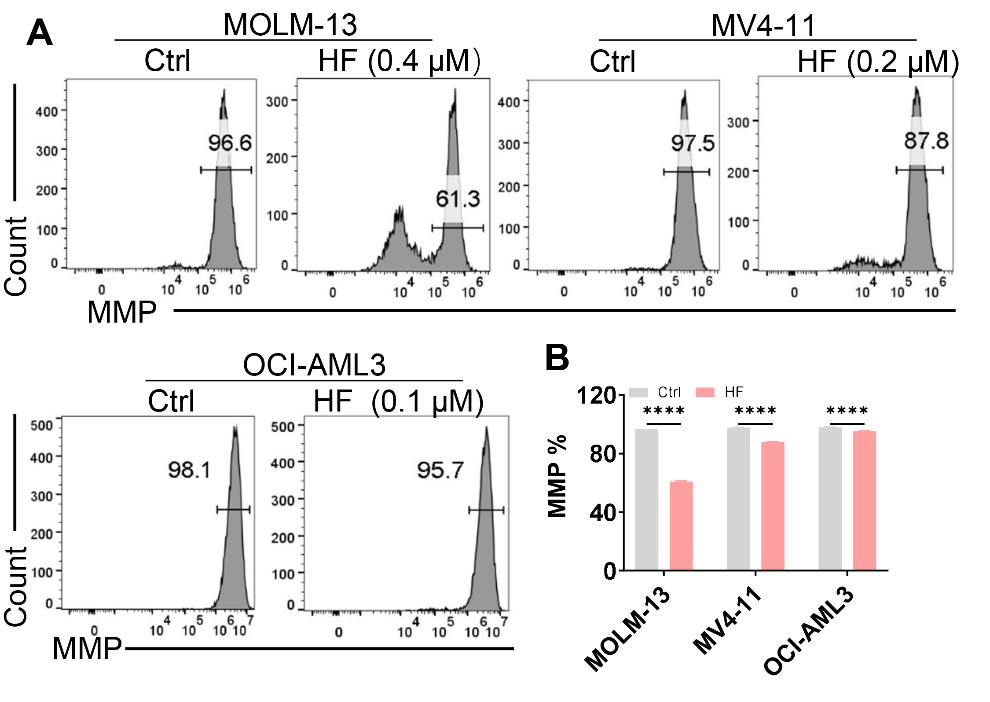


**Figure S3: HF treatment induces the collapse of mitochondrial membrane potential (MMP).** (A and B) MMP was measured by JC-1 labeling of mitochondria through flow cytometry in MOLM-13, MV4-11, and OCI-AML3 cells treated with the indicated concentrations of HF for 24 h. The representative MMP plots (A) and statistical analyses of MMP (B) are shown.


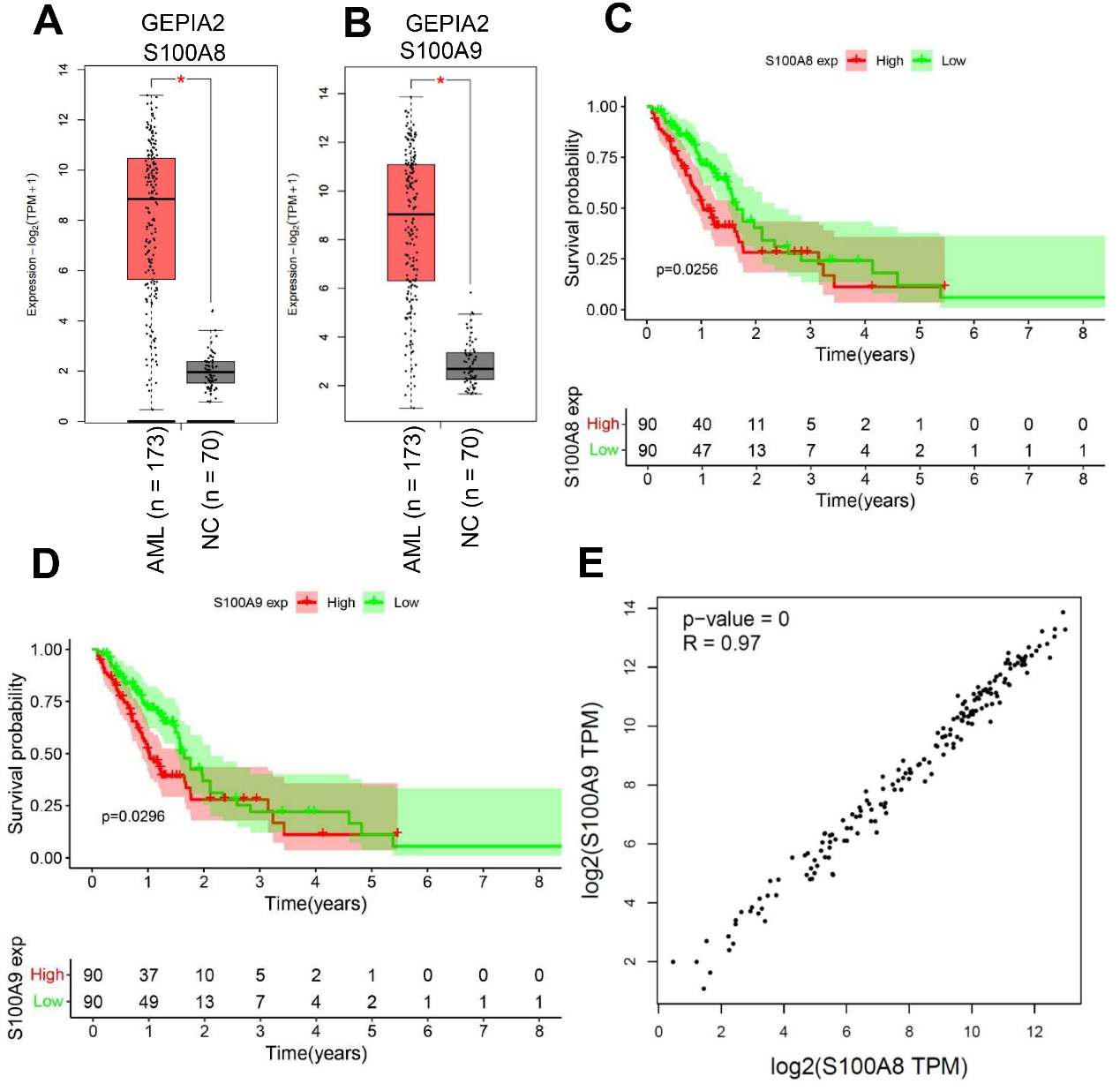


**Figure S4: S100A8 and S100A9 (S100A8/A9) expressions are higher in AML cells than normal controls (NC) and higher expressions of S100A8/A9 predict poor outcome.** (A and B) *S100A8*/*A9* transcript levels were analyzed in AML patients and NC samples using the GEPIA database. (C and D) The predictive outcome was analyzed in AML patients with higher or lower expressions of *S100A8* and *S100A9* using the BEAT AML database. (E) A positive correlation between the expression levels of *S100A8* and *S100A9* was observed. **P* < 0.05.


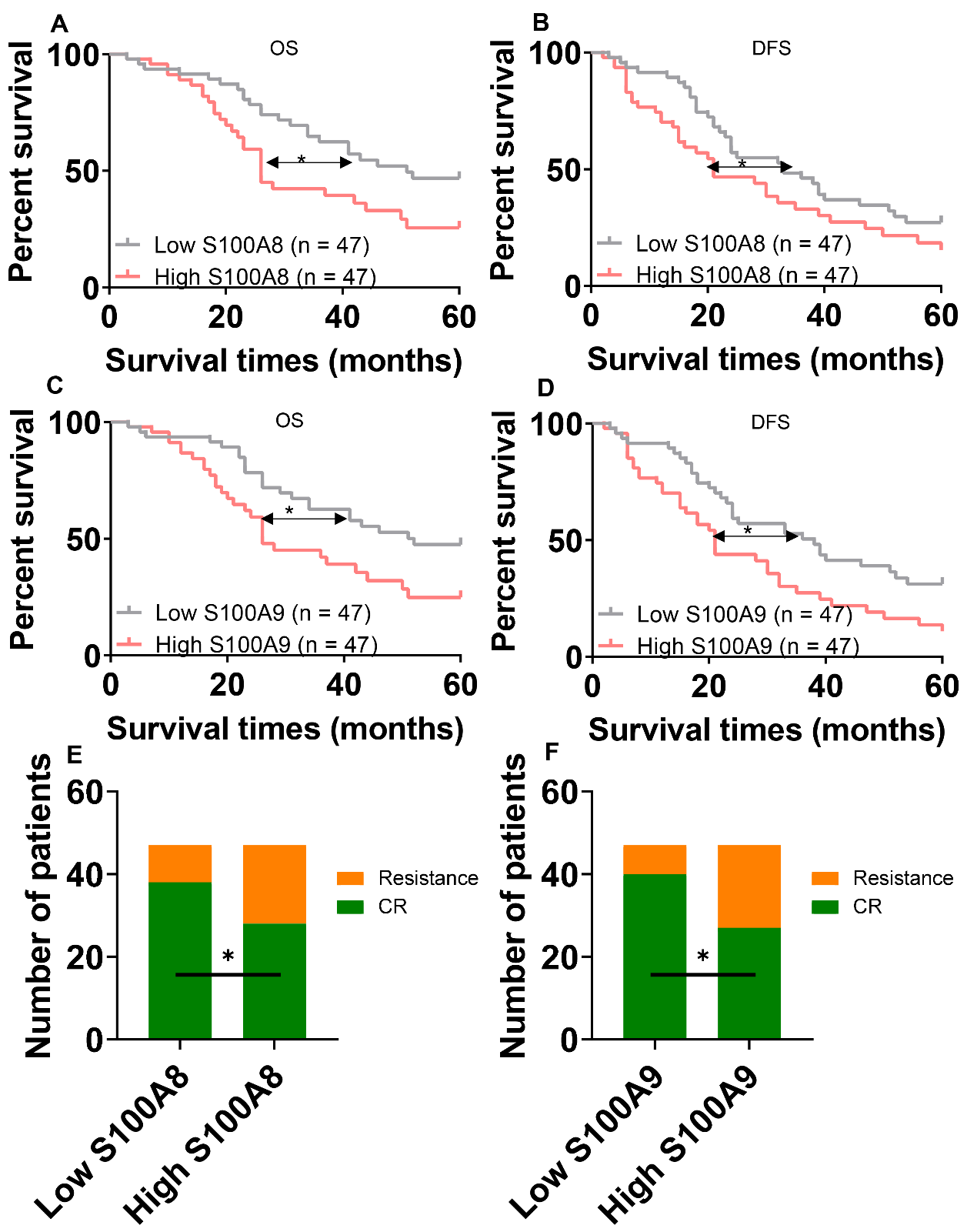


**Figure S5: The clinical relevance of S100A8/A9 expression is analyzed in 94 AML patients received standard induction regimen.** (A and B) Overall survival (OS, A) and relapse-free survival (RFS, B) were analyzed in 94 AML patients with higher (above median) or lower (below median) expressions of *S100A8*. (C and D) OS (C) and RFS (D) were analyzed in 94 AML patients with higher (above median) or lower (below median) expressions of *S100A9*. (E) Complete remission (CR) or resistance response numbers were calculated in AML patients with higher (above median) or lower (below median) expressions of *S100A8*. (F) CR or resistance response numbers were calculated in 94 AML patients with higher (above median) or lower (below median) expressions of *S100A9*.


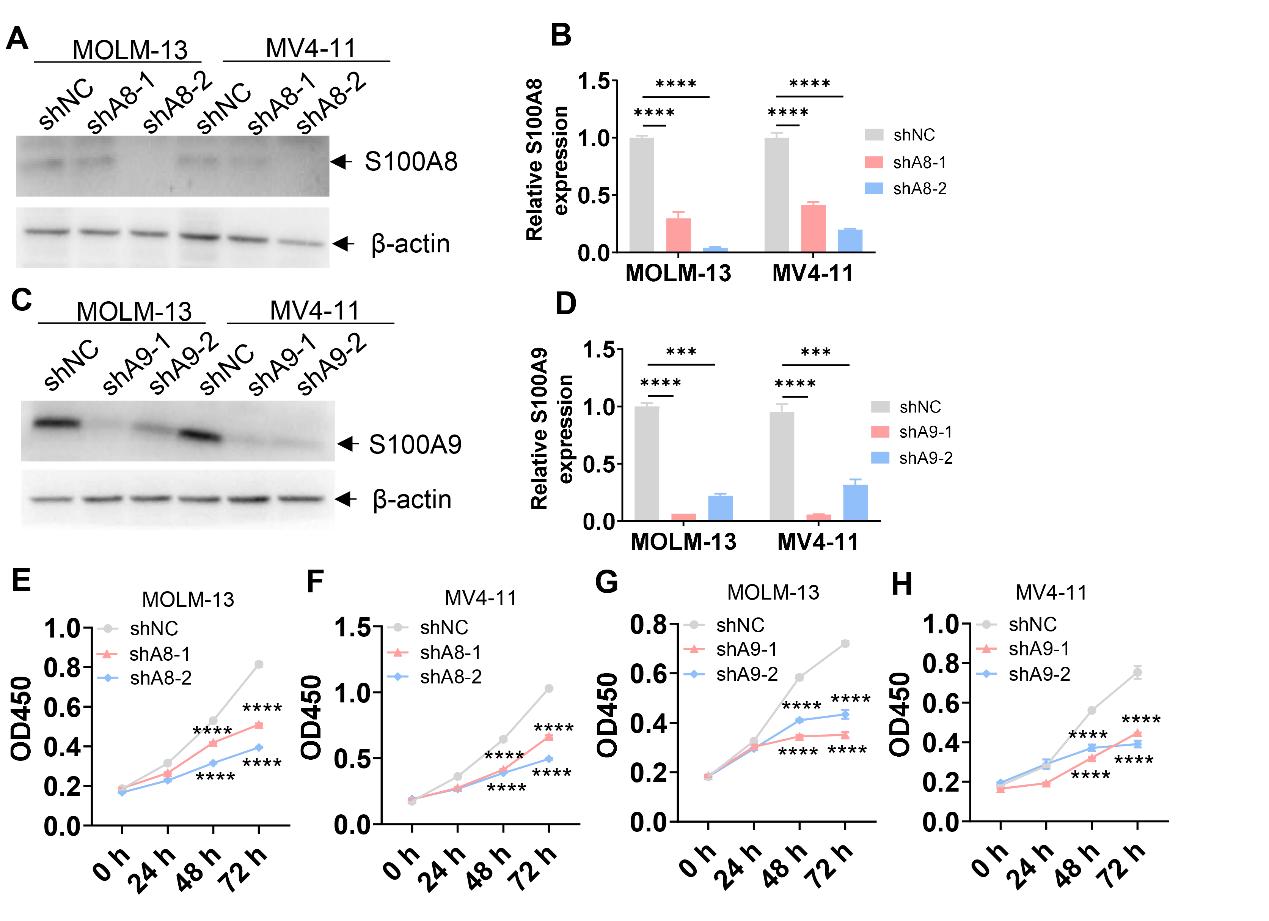


**Figure S6: Knockdown of *S100A8* or *S100A9* suppresses proliferation in AML cells.** (A and B) The protein and transcript levels of S100A8 were measured in MOLM-13 and MV4-11 cells, which were transduced with shRNA for negative control (shNC) or shRNAs targeting S100A8 (shA8-1 and shA8-2) for 48 h and treated with puromycin (1 μg/mL) for an additional 48 h. (C and D) The protein and transcript expressions of S100A9 were measured in MOLM-13 and MV4-11 cells, which were transduced with shNC and shRNAs targeting S100A9 (shA9-1 and shA9-2) for 48 h and treated with puromycin (1 μg/mL) for an additional 48 h. (E and F) Cell proliferation was measured by CCK8 assay in MOLM-13 and MV4-11 cells, transduced with shNC, shA8-1, or shA8-2 for the indicated times. (G and H) Cell proliferation was measured by CCK8 assay in MOLM-13 and MV4-11 cells, transduced with shNC, shA9-1, or shA9-2 for the indicated times. ****P* < 0.001; *****P* < 0.0001 versus shNC.


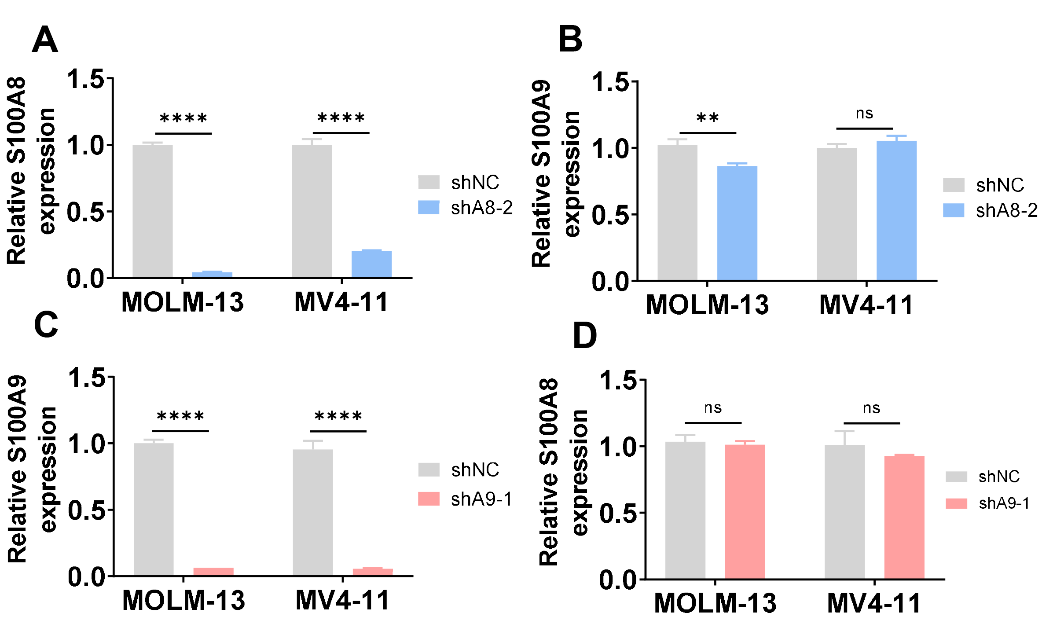


**Figure S7: Specific knockdown of *S100A8* or *S100A9* by shRNAs.** (A and B) The transcript expressions of *S100A8* and *S100A9* were measured in MOLM-13 and MV4-11 cells, which were transduced with shNC or shA8-2 for 48 h. (C and D) The transcript expressions of *S100A8* and *S100A9* were measured in MOLM-13 and MV4-11 cells, which were transduced with shNC or shA9-1 for 48 h.***P* < 0.01; *****P* < 0.0001. ns: not significant.


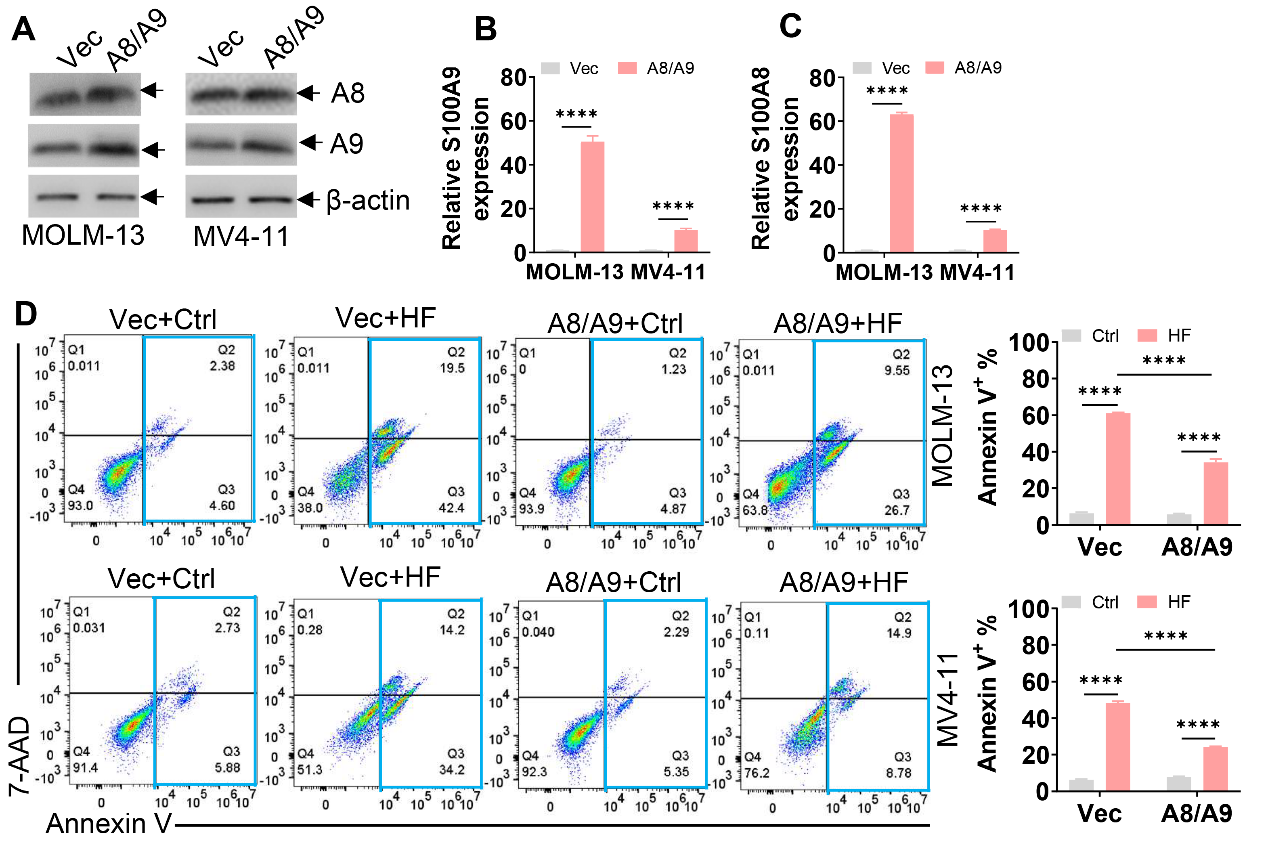


**Figure S8: Overexpression of S100A8/A9 rescues HF-induced apoptosis.** (A–C) The protein and transcript expressions of S100A8/A9 were measured in MOLM-13 and MV4-11 cells, which were overexpressed with S100A8/A9 or negative control (Vec) for 48 h. (D) Apoptosis was measured in MOLM-13 and MV4-11 cells overexpressing S100A8/A9 or Vec, and treated with or without HF (0.4 μM for MOLM-13; 0.2 μM for MV4-11) for 48 h. The representative plots of Annexin V/7-AAD staining (left) and quantification of Annexin V^+^ cells (right) are shown. *****P* < 0.0001.


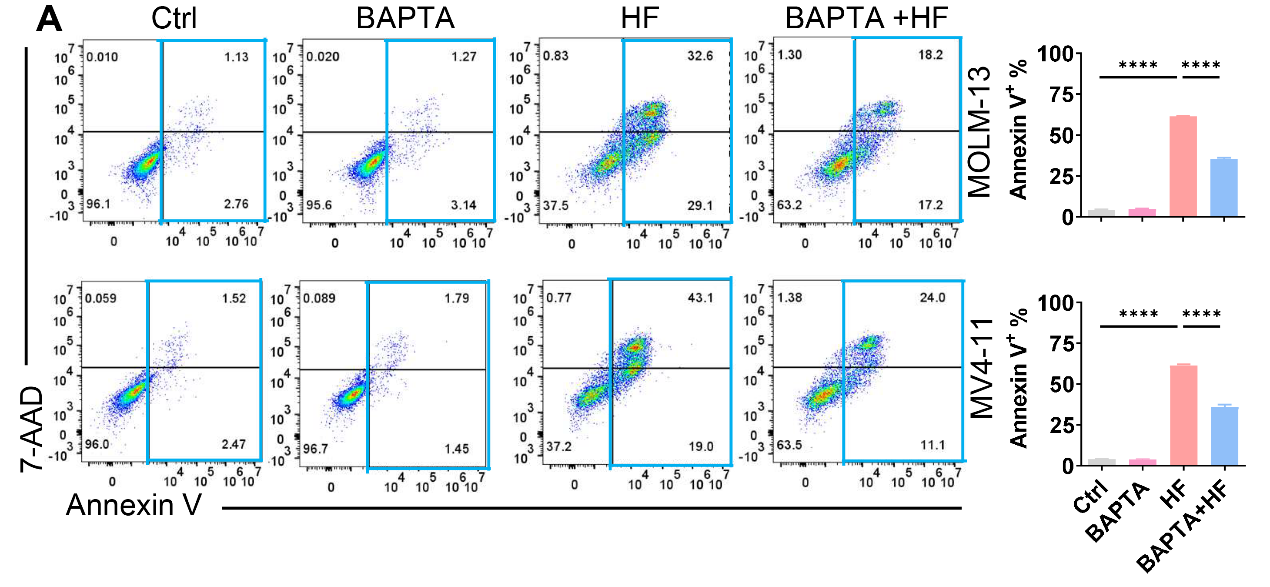


**Figure S9:** Ca^2+^ inhibitor BAPTA-AM (BAPTA) blocks HF-induced apoptosis in AML cells. (A) Apoptosis was measured in MOLM-13 and MV4-11 cells treated with Ctrl, Ca^2+^ inhibitor BAPTA (5.0 μM), HF (0.4 μM for MOLM-13; 0.2 μM for MV4-11), or HF+BAPTA for 48 h. The representative plots (left) and quantification of Annexin V^+^ cells (right) are shown. *****P* < 0.0001.


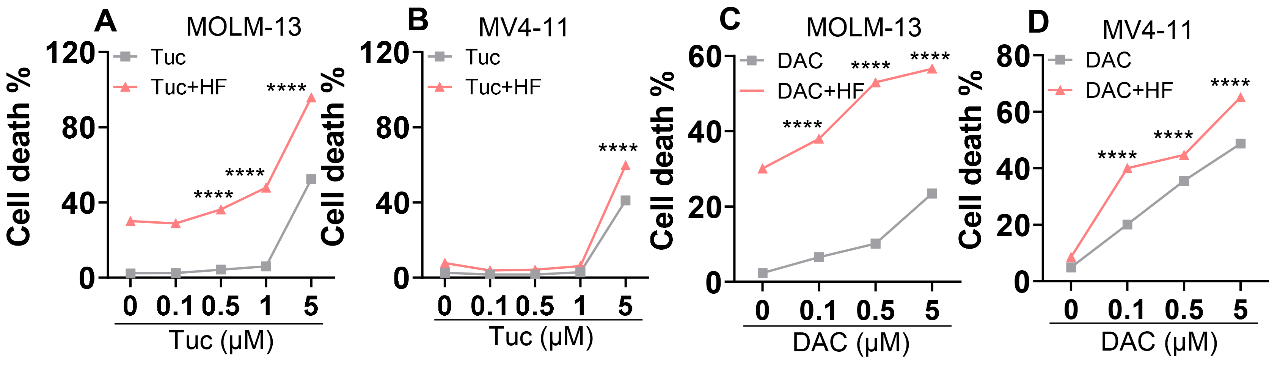


**Figure S10: HF enhances the cytotoxicity of chemotherapeutic drugs in AML cells.** (A and B) Cell death was measured by 7-AAD staining in MOLM-13 and MV4-11 cells treated with HF (0.2 μM for MOLM-13; 0.1 μM for MV4-11), different concentrations of Tucidinostat (Tuc), and combinations of Tuc + HF for 48 h. (C and D) Cell death was measured by 7-AAD staining in MOLM-13 and MV4-11 cells treated with HF (0.2 μM for MOLM-13; 0.1 μM for MV4-11), different concentrations of decitabine (DAC), and combinations of DAC + HF for 48 h. *****P* < 0.0001 versus Tuc or DAC.


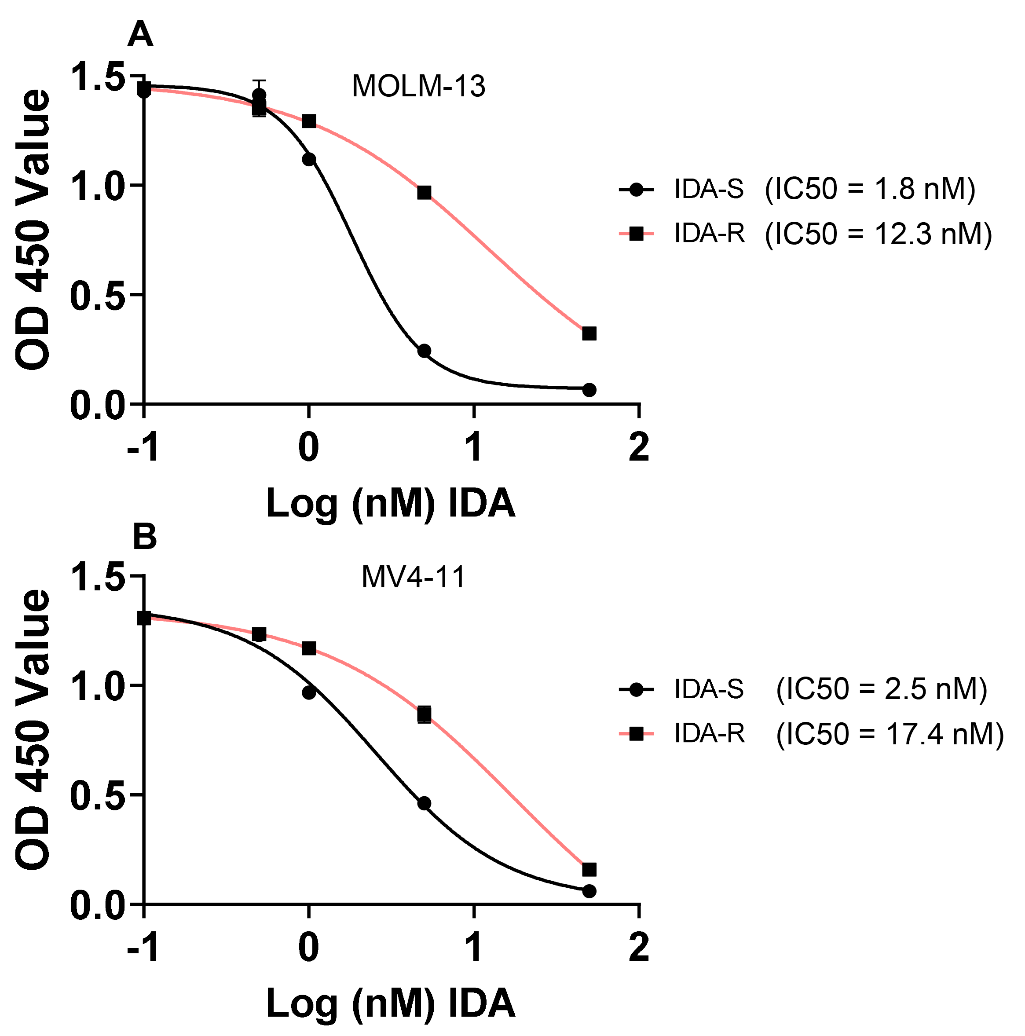


**Figure S11: Half maximal inhibitory concentration (IC50) is measured in MOLM-13-and MV4-11-IDA-resistant (R) and sensitive (S) cells.** (A) OD450 values were measured in MOLM-13-(R) and MOLM-13-(S) cells treated with 0, 0.1, 0.5, 1.0, 5.0, and 50 nM IDA for 24 h, and IC50 was calculated. (B) OD450 values were measured in MV4-11-(R) and MV4-11-(S) cells treated with 0, 0.1, 0.5, 1.0, 5.0, and 50 nM IDA for 24 h, and IC50 was calculated.


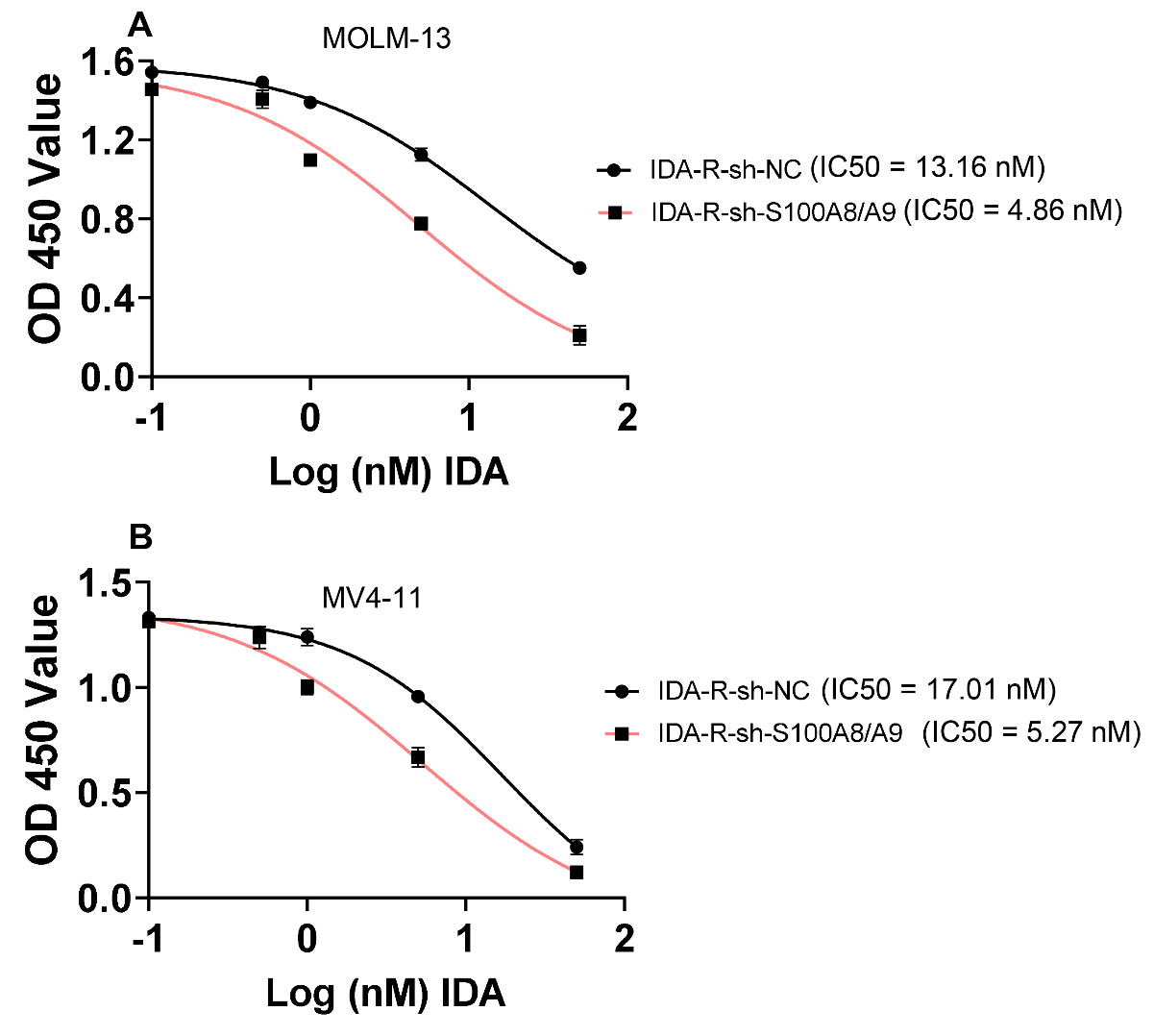


**Figure S12: IC50 is measured in MOLM-13-IDA-R and MV4-11-IDA-R cells transduced with sh-NC or sh-S100A8/A9.** (A) IC50 was calculated in MOLM-13-R cells, which were transduced with shNC or shS100A8/A9 for 48 h, followed by treatment with 0, 0.1, 0.5, 1.0, 5.0, and 50 nM IDA for 24 h. (B) IC50 was calculated in MV4-11-R cells, which were transduced with shNC or shS100A8/A9 for 48 h, followed by treatment with 0, 0.1, 0.5, 1.0, 5.0, and 50 nM IDA for 24 h.


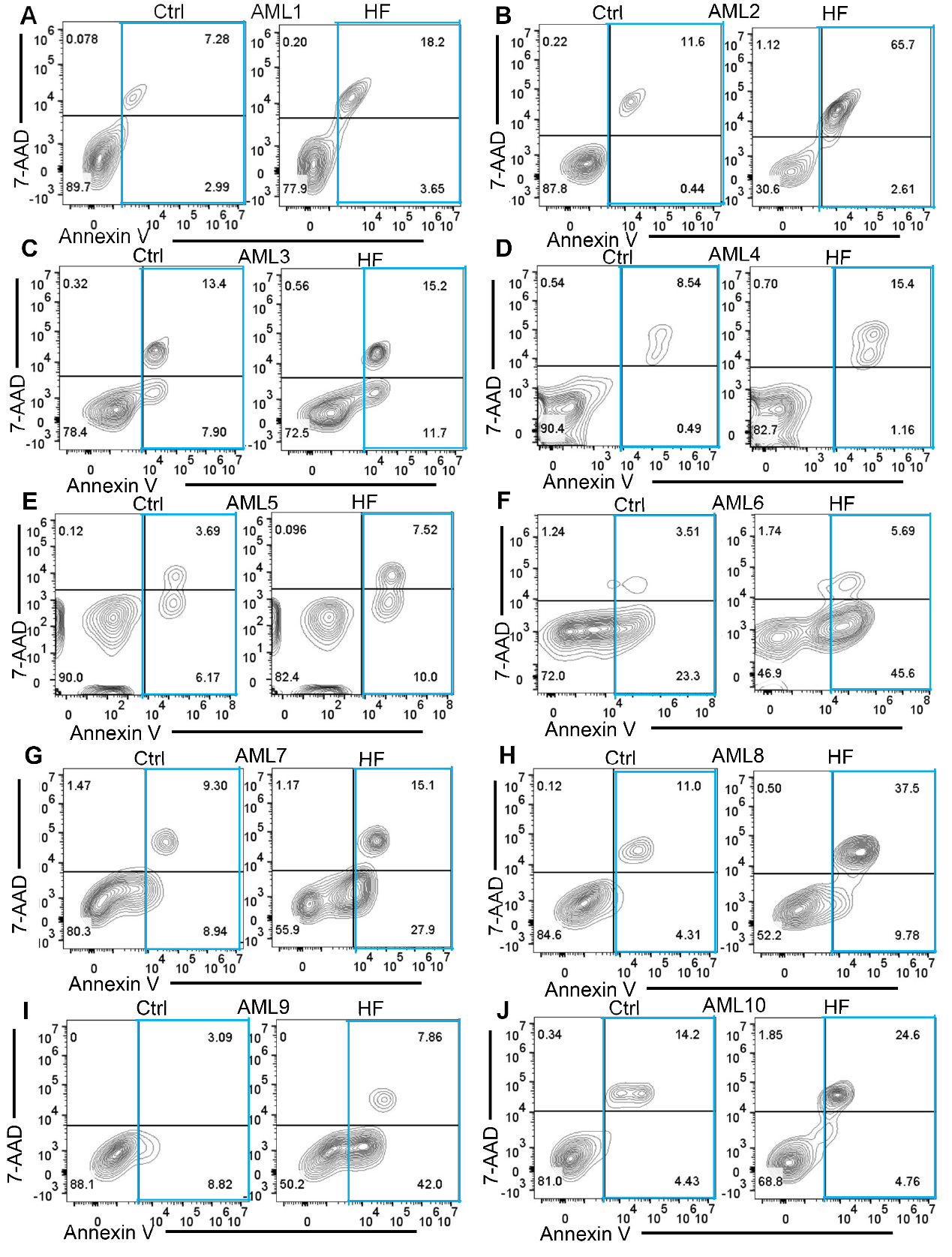


**Figure S13: HF induces apoptosis in primary BM cells from AML patients.** (A–J) Apoptosis was measured by Annexin V/7-AAD staining in BM cells from 10 AML patients (1–10) treated with HF (0.1 μM) for 24 h. The representative plots of Annexin V/7-AAD staining are shown.

**
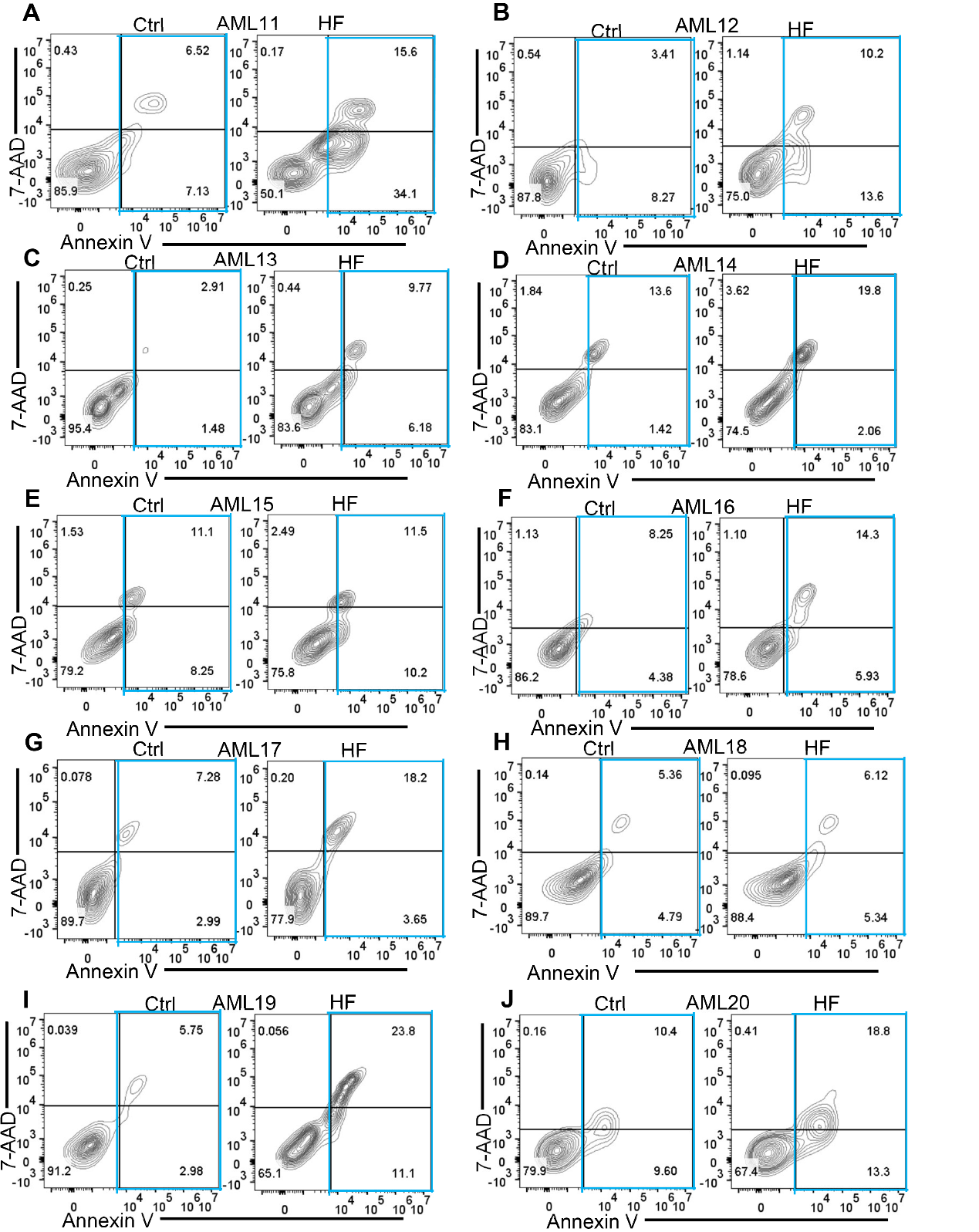
**

**Figure S14: HF induces apoptosis in primary BM cells from AML patients.** (A–J) Apoptosis was measured by Annexin V/7-AAD staining in BM cells from 10 AML patients (11–20) treated with HF (0.1 μM) for 24 h. The representative plots of Annexin V/7-AAD staining are shown.

**
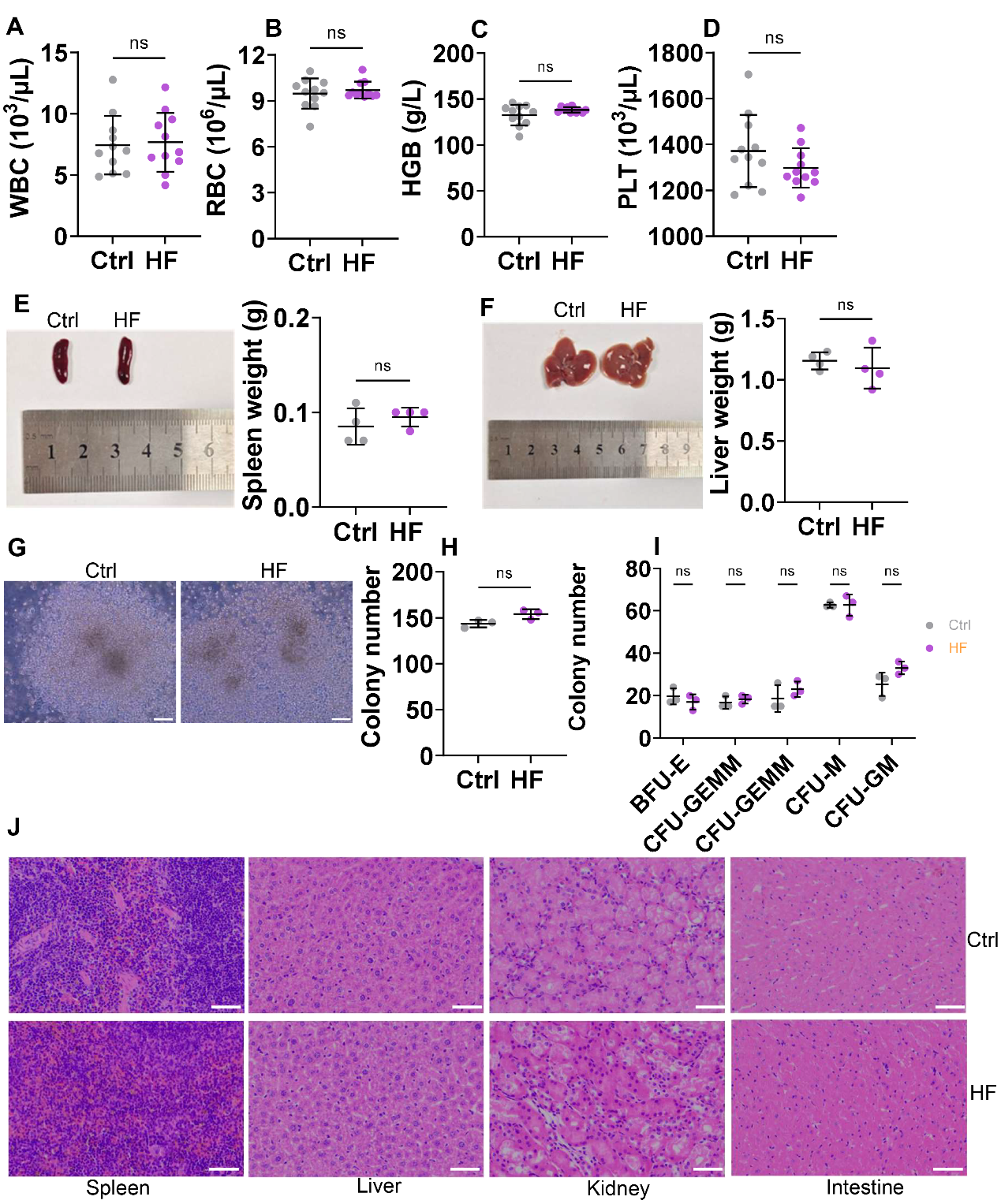
**

**Figure S15:** **HF has little effect on normal hematopoiesis.** Two-month-old wild-type C57/B6 mice were treated with HF or Vehicle (Ctrl) for two weeks. After one month from the last injection, PB and BM cells were extracted for the subsequent analysis. (A–D) Blood count analysis was performed in the PB from Ctrl (n = 11) and HF (n = 11)-treated mice. A for White blood cell (WBC) count; B for red blood cell (RBC) count; C for hemoglobin (HGB) levels; D for platelet (PLT) count. (E and F) Spleen and liver tissues were obtained from Ctrl and HF-treated mice (n=4 for spleen and liver of each group), and the weights were measured. Representative images (left) and statistical analysis of liver and spleen weights (right) are shown. (G–I) BM c-Kit^+^ cells were isolated from Ctrl and HF-treated mice (n=3 for each group) and plated in methylcellulose medium for 10 days to count colony (2×10^3^/dish for each group). Shown are the representative images (G) and statistical analysis of colony numbers (H) and the classification of burst-forming unit-erythroid (BFU-E), colony-forming unit-granulocyte, erythroid, macrophage, and megakaryocyte (CFU-GEMM), colony-forming unit-granulocyte (CFU-G), colony-forming unit-macrophage (CFU-M), and colony-forming unit-granulocyte and macrophage (CFU-GM) (I). Bar scales represent 200 μm. (J) HE staining was performed for histological examination in the liver, spleen, heart, kidney, and intestine from HF-treated or untreated mice. ns: not significant.

**Table S1. The sequences of primers for qRT-PCR and construction of plasmids**

^#^Primers for construction of plasmids

| Genes | Sequences |
| --- | --- |
| GAPDH-F | 5′-CCG GGA AAC TGT GGC GTG ATG G-3′ |
| GAPDH-R | 5′-AGG TGG AGG AGT GGG TGT CGC TGT T-3′ |
| S100A9-F | 5′-GGTCATAGAACACATCATGGAGG-3′ |
| S100A9-R | 5′-GGCCTGGCTTATGGTGGTG-3′ |
| S100A8-F | 5′-ATGCCGTCTACAGGGATGAC-3′ |
| S100A8-R | 5′-ACTGAGGACACTCGGTCTCTA-3′ |
| S100A4-F | 5′-GAT GAG CAA CTT GGA CAG CAA-3′ |
| S100A4-R | 5′-CTG GGC TGC TTA TCT GGG AAG-3′ |
| S100A12-F | 5′-AGC ATC TGG AGG GAA TTG TCA-3′ |
| S100A12-R | 5′-GCA ATG GCT ACC AGG GAT ATG AA-3′ |
| eIF2α-F | 5′-TGG TGA ATG TCA GAT CCA TTG C-3′ |
| eIF2α-R | 5′-TAG AAC GGA TAC GCC TTC TGG-3′ |
| S100a9-F (mouse) | 5′-ATA CTC TAG GAA GGA AGG ACA CC-3′ |
| S100a9-R (mouse) | 5′-TCC ATG ATG TCA TTT ATG AGG GC-3′ |
| S100a8-F (mouse) | 5′-AAA TCA CCA TGC CCT CTA CAA G-3′ |
| S100a8-R (mouse) | 5′-CCC ACT TTT ATC ACC ATC GCA A-3′ |
| β-actin-F (mouse) | 5′-GGC TGT ATT CCC CTC CAT CG-3′ |
| β-actin-R (mouse) | 5′-CCA GTT GGT AAC AAT GCC ATG T-3′ |
| ^#^sh-S100A8#1 | 5′-CCA CAA GTA CTC CCT GAT AAA-3′ |
| ^#^sh-S100A8#2 | 5′-TCA ACA CTG ATG GTG CAG TTA-3′ |
| ^#^sh-S100A9#1 | 5′-CCT GGA CAC AAA TGC AGA CAA-3′ |
| ^#^sh-S100A9#2 | 5′-CGC AAC ATA GAG ACC ATC ATC-3′ |
| ^#^sh-eIF2α#1 | 5′-GAA GTA CTC ATT AAT AAT ATT A -3′ |
| ^#^sh-eIF2α#2 | 5′-TGG ATA GTT TAG ATT TGA ATG A-3′ |
| ^#^sh-eIF2α#3 | 5′-CGG GAA GTA CTC ATT AAT AAT A-3′ |
| ^#^LVX-S100A8-F | 5′-AGG ATC TAT TTC CGG TGA ATT CAT GTC TCT TGT CAG CTG TCT-3′ |
| ^#^LVX-S100A8-HA-R | 5′-GGG AGG GAG AGG GGC GGG ATC CCT AAG CGT AAT CTG GAA CAT CGT ATG GGT ACT CTT TGT GGC TTTC-3′ |
| ^#^LVX-S100A9-F | 5′-AGG ATC TAT TTC CGG TGA ATT CAT GAC TTG CAA AAT GTC GCA-3′ |
| ^#^LVX-S100A9-HA-R | 5′-AGG GAG AGG GGC GGG ATC CTT AAG CGT AAT CTG GAA CAT CGT ATG GGT AGG GGG TGC CCT CCC CGA G-3′ |

**Table S2. The clinical characteristics of 94 AML patients for outcome analysis**

| Characteristic | All patients  N (%) |  |
| --- | --- | --- |
| Overall | 94 |  |
| Gender |  |  |
| Male | 48 (51.0) |  |
| Female | 46 (49.0) |  |
| Age (range) | 53 (21-73) |  |
| FAB subtype |  |  |
| M0-M1 | 10 (10.6) |  |
| M2 | 13 (13.8) |  |
| M4 | 35 (33.9) |  |
| M5 | 36 (35.1) |  |
| Cytogenetics |  |  |
| Normal karyotype | 38 (40.4) |  |
| Complex karyotype | 21 (22.3) |  |
| t(8;21) | 14 (14.8) |  |
| Inv(16) | 10 (10.6) |  |
| t(11q23) | 9 (9.5) |  |
| Not available | 2 (2.1) |  |
| Genetic mutation |  |  |
| WT1 mutation | 11 (11.7) |  |
| FLT3 mutation | 12 (12.7) |  |
| CEBPA mutation | 16 (17.0) |  |
| MLL-PTD mutation | 2 (2.1) |  |
| TET2 mutation | 10 (10.6) |  |
| NPM1 mutation | 5 (5.3) |  |
| KIT mutation | 8 (8.5) |  |

**Table S3. Detailed characteristics of 20 AML patients for HF treatment**

| N | Gender | Age (year) | Diagnosis | FAB type | Blasts% | Cytogenetics | Molecular |
| --- | --- | --- | --- | --- | --- | --- | --- |
| 1 | F | 49 | De novo | M1 | 84 | 46, XX | FLT3-TID, |
| 2 | F | 37 | De novo | M2 | 94 | 46, XX, t(8;21)  (q22;q22) | AML1-ETO, WT1 |
| 3 | M | 51 | R/R | M5 | 90 | 46,XY,del(11)(q22) | TP53, NRAS |
| 4 | F | 63 | De novo | M5 | 65 | 46, XX | WT1 |
| 5 | M | 74 | De novo | M4 | 71 | 45,XY,-7,add(8)(p11.2) | CEBPA, IDH2 |
| 6 | M | 36 | R/R | M5 | 83 | 46,XY,der(9)t(9;11)(p22:q23) | WT1 |
| 7 | F | 86 | De novo | M4 | 61 | 46, XX | TP53, IDH2 |
| 8 | F | 50 | De novo | M3 | 73 | 46,XX,t(15;17)(q22;q21) | PML-RARα |
| 9 | F | 52 | De novo | M2 | 80 | 46, XX | WT1, GATA2 |
| 10 | M | 69 | De novo | M2 | 76 | 46, XY, t(8;21)  (q22;q22) | CEBPA |
| 11 | F | 61 | R/R | M4 | 69 | 46, XX | FLT3-ITD |
| 12 | M | 57 | De novo | M3 | 75 | 46, XY, t(15;17)(q22;q21) | PML-RARα |
| 13 | M | 80 | De novo | M5 | 51 | 46, XY | RUNX1 |
| 14 | M | 58 | De novo | M5 | 58 | 47, XY, +21 | PTPN11 |
| 15 | F | 74 | De novo | M4 | 83 | Complexed karyotype | FLT3-ITD, |
| 16 | M | 66 | De novo | M2 | 77 | 46, XY, t(8;21)  (q22;q22) | AML1-ETO, c-KIT |
| 17 | M | 60 | R/R | M4 | 74 | 45, XY, -7, | FLT3-TKD |
| 18 | F | 78 | De novo | M5 | 83 | 46, XX | CEBPA |
| 19 | F | 48 | De novo | M2 | 85 | 46, XX | DNMT3A |
| 20 | F | 51 | De novo | M4 | 78 | Complexed karyotype | CEBPA, WT1 |
